# Water as the reaction medium in organic chemistry: from our worst enemy to our best friend

**DOI:** 10.1039/d0sc06000c

**Published:** 2021-02-12

**Authors:** Margery Cortes-Clerget, Julie Yu, Joseph R. A. Kincaid, Peter Walde, Fabrice Gallou, Bruce H. Lipshutz

**Affiliations:** Chemical & Analytical Development Novartis Pharma AG 4056 Basel Switzerland mcortes@chem.uesb.edu; Department of Materials, ETH Zurich Zurich Switzerland; Department of Chemistry & Biochemistry, University of California Santa Barbara California 93106 USA

## Abstract

A review presenting water as the logical reaction medium for the future of organic chemistry. A discussion is offered that covers both the “on water” and “in water” phenomena, and how water is playing unique roles in each, specifically with regard to its use in organic synthesis.

## Abstract 2

A review that highlights water as the logical reaction medium in which organic chemistry can be practiced. The key roles that water can play in directing reaction outcomes, including impacting mechanistic features, are discussed using selected examples.

## Graphical abstract

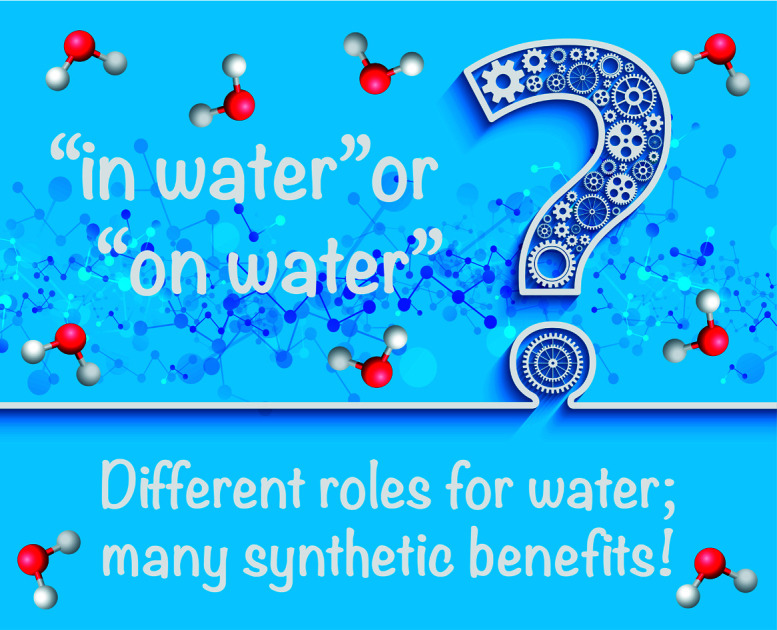

## 1. Introduction

Although water is Nature's solvent, it has long been regarded by most organic chemists, at least until recently, as their worst enemy. From the halls of academia specializing in courses on introductory organic chemistry to multi-kilo labs still the domain of process chemists worldwide, all are taught that the presence of water in so many fundamental organic reactions should be avoided. Historically, the paradigm that “like dissolves like”, implying that dissolution is a prerequisite for high reaction conversion, led to the obvious conclusion that water is a “no-go”. This notion may have arisen from the observation that for some catalysts, reagents, and/or reaction conditions, there is a definite element of moisture sensitivity. Thus, organic solvents, and when necessary, *very dry* organic solvents, have always been the norm, with most subsequent developments made with this in mind. However, toxicity issues such as mutagenicity, teratogenicity, carcinogenicity, and/or reprotoxicity can be ascribed to many of these same solvents. The risk to operators in the plant due to flammability, explosivity, and exposure, in general, is not trivial, whether arising from their industrial applications, transportation, and/or storage. Their impact on the environment must not be overlooked either. Volatile organic compounds (VOCs) such as solvents can contribute to smog, air pollution, ground-level ozone production and yes, climate change. The persistence of chlorinated solvents in soils and aquatic environments represents yet another non-negligible environmental threat.^1^ For these reasons, regulations are becoming increasingly severe regarding production and use of organic solvents, forcing chemists to find greener and safer alternatives. While the Montreal Protocol^2^ has aimed to control usage of nearly 100 man-made ozone-depleting substances since 1987, the Registration, Evaluation, Authorization and Restriction of Chemicals (REACH)^3^ regulation has more recently been adopted in Europe, looking to protect both human health and the environment from the risks posed by chemicals. Even big oil companies (*e.g.*, British Petroleum), otherwise the foundation of our petroleum-based economy and suppliers of so many of our chemicals and especially, organic solvents, have made it clear that “oil has peaked”; that it will be on the decline for the next several decades. As Bernard Looney, CEO of BP recently stated: “We're pivoting from being an international oil company to an integrated energy company”.^4^ Isn't the “handwriting on the wall”?

On the other hand, water as the main, if not exclusive reaction medium for organic transformations represents a safe, non-toxic, cheap, and environmentally friendly alternative. Since the seminal work of Breslow in 1980,^5^ and despite previous and current dogma to the contrary, a large variety of organic reactions have been proven to take place in aqueous media, sometimes with outstanding enhancements, such as faster reaction rates and greater selectivities compared to results obtained using classic organic solvent-based systems. Indeed, water possesses unique physical and chemical properties; it is the medium chosen by Nature in which all of life operates, playing by rules determined over billions of years. Is it, therefore, surprising that new and unexpected experimental results are being discovered in this medium, a medium that has been essentially overlooked throughout the 150–200 years of modern organic chemistry?

This review is not meant to be an exhaustive cataloging of existing literature on chemistry in water; rather, the intent is to cover selected mechanistic aspects that involve, and may even favor, use of water in organic transformations. Depending on the conditions, water can be regarded as a medium, where, for example, no solvation of the reaction components takes place (*i.e.*, processes “on water”). Alternatively, water can be present within the medium (*i.e.*, “with water”), or as the medium in which there are additives that help solubilize the otherwise water-insoluble educts, catalysts, *etc.* (*i.e.*, “in water”).^6^ Given the accent on water as reaction medium, neither phase transfer catalysis nor aqueous biphasic catalysis is discussed herein. The former relies, by definition, on the use of a non-miscible organic solvent. One underlying theme throughout this review is that of using water in place of organic solvents, which is the case with both “on water” and “in water” descriptors; there is only a single example herein showcasing “in water with organic solvents”. Indeed, perhaps subliminally, the notion of replacing these traditional, waste-generating solvents will appeal to the reader for further consideration. The latter area involving water-soluble catalysts and slightly soluble educts, so commonly used in the chemical industry on huge scales, is also overlooked. This review targets industries that have been hesitant to consider water as the reaction medium, especially the pharmaceutical area where the organic solvents used routinely lead to the majority of organic waste produced by the entire chemical enterprise. Moreover, the scales and time frame under which those in the fine chemical area operate are notably different; here, the accent must be not only on sustainability, but also on efficiency, and a switch to this “new” medium, water, offers both. Admittedly, there are aspects to this evolving area of chemistry in water that are poorly understood, if understood at all; but these, hopefully, will be recognized as providing opportunities for discovery while simultaneously assisting the practitioner to contribute to our inevitable move away from a petroleum-based discipline, following Nature's lead.

We shall focus particularly on the properties of water that make it special; indeed, a unique reaction medium with classifications of reactions such as those “on water”, and those “in water” featuring soft and dispersed interface-rich aqueous systems^7^ (*e.g.*, soft matter associated with “micellar catalysis”). A selection of applications illustrating the mechanistic implications of, and roles played by, water and its benefits on the reactivity and selectivity associated with various reactions will also be presented.

## 2. Classification of reactions using water as the reaction medium

### (a) Early work

**Fig. 1:**
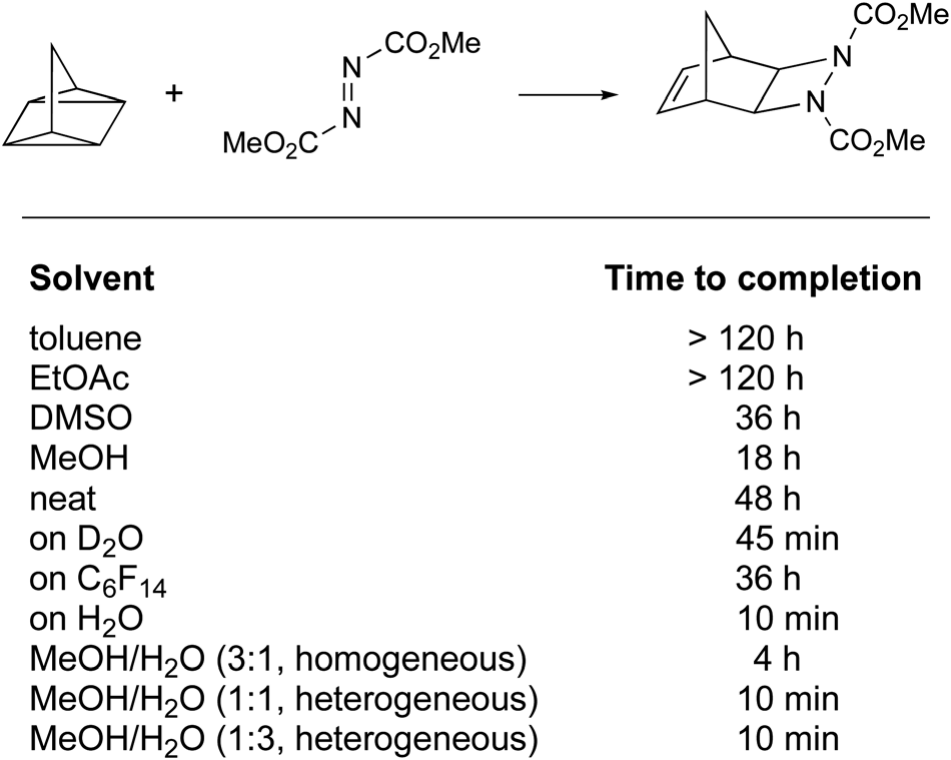
Cycloaddition reaction accelerated “on water” (Sharpless, 2005).^8^

The definition of “on water” reactions has been a topic of discussion over the last 15 years. The term, introduced by Sharpless in 2005, was first described as leading to “substantial rate acceleration when insoluble reactants are stirred in aqueous suspension”.^8^ This statement highlighted two parameters: the rate of the reaction and lack of substrate solubility in water. In this study, a number of reactions were presented, including a [2σ + 2σ + 2π] cycloaddition performed “on water” at molar concentrations. All were accelerated when only water was used as the “solvent”, as opposed to polar and non-polar organic solvents, illustrated by the reaction of quadricyclane with dimethyl azodicarboxylate (Fig. 1).

The “on water” reaction reached completion after 10 min, while 48 h and more than 18 h were required using neat conditions and in organic solvents, respectively. The gradual addition of methanol to water was of no consequence, as long as reaction heterogeneity was conserved. Above a critical concentration of methanol leading to a homogeneous environment, the reaction time was extended to 4 h. While heterogeneity seems to play a role, it is important to notice that the reaction performed in perfluorohexane was not faster than in other organic solvents (reaction time: 36 h). Other parameters need to be considered; *e.g.*, results under homogeneous conditions highlighted that hydrogen bonding and polarity might play a role as well (MeOH > DMSO > toluene). Most cases of intermolecular reactions studied involve liquids or oils, since solids present additional issues of “mixing” during “on water” reactions.

**Fig. 2:**
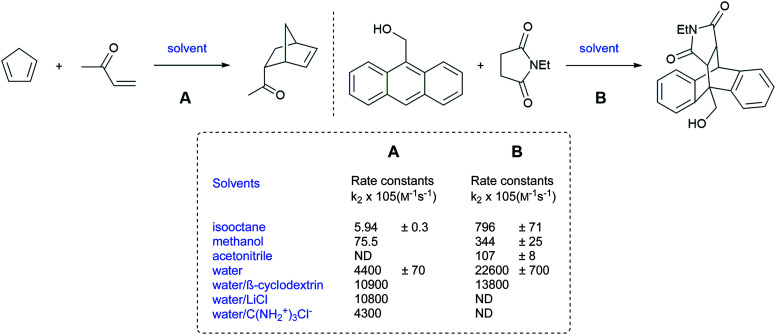
Diels–Alder reactions accelerated by “on water” conditions (Breslow).^5^

Those results provided foreshadowing as to the as yet poorly understood but synthetically advantageous use of water as a reaction medium in organic chemistry. Despite running at low concentrations (mM or less), Rideout and Breslow postulated that the acceleration of the Diels–Alder reaction between cyclopentadiene and butenone, in water, was due to the hydrophobic effect. Indeed, the reaction rate, in water, was 58-fold and more than 700-fold higher than in methanol and hydrophobic solvents, respectively (Fig. 2).^5^ By contrast, the reaction between anthracene-9-carbinol and ethyl maleimide showed higher rates in non-polar hydrocarbon solvents than in methanol. Water, however, remained the best medium, leading the authors to conclude that the polarity of the medium was not the explanation here, but rather due to a hydrophobic effect. Moreover, the salting-out effect of LiCl, by further decreasing the solubility of the organic partners in water, led to even faster rates. Moreover, the presence of guanidinium chloride served to reduce hydrophobic interactions leading to slower reactions, thereby ruling out the theory that dissolution of the organic reactants was essential.

These early examples performed with water as the sole medium differ insofar as several reaction parameters are concerned:

(1) Sharpless reported a [2σ + 2σ + 2π] cycloaddition under heterogeneous conditions.^8^

(2) Breslow reported a Diels–Alder cycloaddition using homogeneous conditions.^5^

(3) Breslow also reported a different behavior depending on the nature of the substrates.^5^

(4) Both highlighted the hydrophobic effect.

### (b) Classification: what does the literature say?

In order to explain reaction rate enhancements obtained “on water”, most models assume involvement of purely water-insoluble, hydrophobic solutes, whether liquids, gases, or solid spheres. Experimentally, the actual polarity of substrates can be more difficult to assess. In some cases, the presence of H-bond donor or acceptor functional groups such as ketones, amines, or alcohols, can all follow different rules. Additionally, the type of reaction and the geometry of the associated transition state for these can lead to varying mechanisms, as well as varying reaction outcomes.

Thus, while important, a clear distinction between “on water” and “in water” is not easy to draw. While reactants seem macroscopically suspended in so-called “on water” reactions, reports indicate that the reaction might actually be happening “in water”, where limited amounts of dissolved starting materials may be present. Butler and Coyle enriched the initial definition of “on water” conditions from Sharpless as follows: “…applies to organic reactions that occur between water insoluble reactants at the interface of the bulk liquid water phase that contains no additives. It does not apply to reactions in the presence of very small quantities of water, such as hydrated salts, or involving catalysts”.^9^ Part of this definition, however, was recently disputed by Kobayashi (*vide supra*).^10^ Nonetheless, Butler and Coyle nuanced this claim by introducing a classification based on substrate solubility and the location in which the transition state occurs. The reaction is considered to take place “in water” if the solubility of substrates is >0.01 mol L^−1^, and the transition state is in bulk water. “On water” conditions apply to substrates with solubility lower than 10^−5^ mol L^−1^ and with a transition state occurring on the organic side of the interface. Finally, for reactants with intermediate solubilities, both modes of reaction are likely to occur simultaneously. For the “in water” scenario, the hydrophobic effect and the cohesive energy density are the key factors leading to a tighter transition state, thus a faster reaction rate. In the “on water” scenario, *trans* H-bonding, or even acid catalysis, at the interface is most likely the predominant parameter that accelerates reactions. These situations are summarized in Table 1.

**Table 1:** Parameters defining the reaction mode in water

Solubility range (mol L^−1^)	10^−2^	10^−3^–10^−5^	<10^−5^
Droplet size	Nanometer (nm)		Millimeter (mm)
Reaction mode	Mainly “in water”	Mainly “on water”	“on water”
	Some “on water”	Some “in water”	
Water solubility	Slightly soluble	Sparingly soluble	Very sparingly soluble
Operating mechanism	Hydrophobic normal H-bonding	Hydrophobic *trans* H-bonding	*trans* H-bonding
	*trans* H-bonding		

**Fig. 3:**
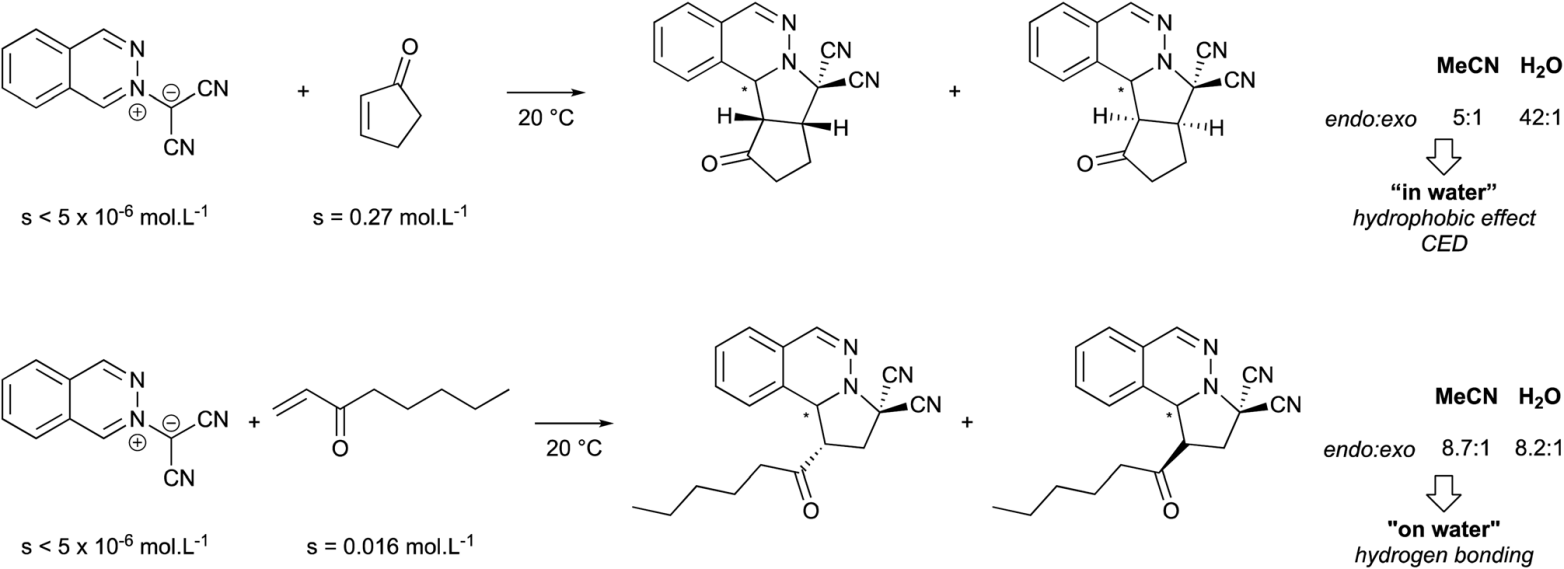
Substrate solubility dependence guiding the reaction mode in water (CED = Cohesive Energy Density).

The following examples illustrate how macroscopic appearance can be misleading (Fig. 3). While both reactions are heterogeneous, the first involves a slightly soluble 2-cyclopentadien-1-one, that is able to carry the insoluble dipole reactant into water. The mechanism led to a higher *endo* : *exo* ratio “in water” (42 : 1) than in acetonitrile (5 : 1) due to a smaller transition state volume. The second example involves two very sparingly soluble reactants, resulting in an “on water” reaction mechanism. In this case, *trans* hydrogen bonding accelerates cycloaddition, which has no impact on both stereoselectivity and the *endo* : *exo* ratio.

In their latest review, Kitanosono and Kobayashi proposed to collectively categorize all reactions using water as the reaction medium, with or without use of catalysts, as “in water”.^10^ They classified each into three types, with seven sub-categories (Table 2). “Type I” is an aqueous-phase reaction, where all reactants are soluble in water. Depending on the solubility of the catalyst (if any), the reaction is considered to be “type Ia” (soluble) or “type Ib” (insoluble). In “type III” reactions, the lipophilic reactants aggregate to form a suspension. If the catalyst is soluble in water, the reaction is a “type IIIa”, while the reaction is a “type IIIb” if the catalyst is soluble in the lipophilic reactants phase. If the catalyst is soluble in neither, the reaction is a “type IIIc”. Lastly, “type II” reactions characterize reactions in water in the presence of surfactants to form a micellar environment. In “type IIa”, the catalyst is soluble in water, while in type IIb it is water-insoluble. The pros and cons of using one method over another are summarized in Table 3.

**Table 2:** Kobayashi's classification of catalytic reactions performed in water depending on substrate/catalyst solubilities

Type	Surfactant	Substrates soluble in water	Catalyst soluble in water	Interfacial reaction site
Ia	No	Yes	Yes	—
Ib	No	Yes	No	Catalyst–water
IIa	Yes	—	Yes	Micelle surface
IIb	Yes	—	No	Micelle surface
IIIa	No	No	Yes	Substrates–water
IIIb	No	No	No	Substrates–water
IIIc	No	No	No	Catalyst–substrates–water

**Table 3:** Summary of pros and cons of reactions using water as the medium

	“In water”	“On water”	Micellar catalysis
Volume variation of the transition state	Negative	Negative	Negative or positive
Substrate solubility in water	Yes	No	Better conversion if insoluble
Pros	High stereoselectivities	• Direct filtration as only work-up; washings to remove potential excess of chemicals and side-products	• Option to extract or to precipitate product
			• Versatile in terms of reactions and substrates
			• High local concentration leading to higher yields
			• Low expected catalyst loading required
			• Mild conditions limiting by-product impurities
Cons	• Limitations on size and solubility of substrates	• Nature of the functional groups (*trans* H-bonding needed)	• Residual surfactant contamination
	• Limited scope of transformations and scope of reactants	• Potential oiling, gumming (difficulties to scale-down, -up)	
	• Extractive work-up required	• High temperature likely to be required to favor exchanges, leading to reduced selectivities	
		• Likely very slow reactions	

Clearly, notwithstanding a positive outcome, different mechanisms are involved. A tremendous amount of research has been directed towards explaining the origins of such accelerations. While the hydrophobic effect and enforced hydrophobic interactions may both be important factors, rationalizing this phenomenon solely by considering the hydrophobic component between substrate(s) and water, without consideration as to how water molecules respond to “intruders” may be an over-simplification. Water is hardly a simple “solvent”; rather, it is a non-inert medium with extraordinary properties. It has both a high cohesive energy density and dielectric constant, and yet remains liquid at ambient pressure; truly unique features. Identification of these multi-faceted parameters and their impact on both the aqueous and lipophilic phases will lead to an enhanced understanding as to which of these, or both, can be used to synthetic advantage on a case-by-case basis.

## 3. Mechanistic aspects

This section aims to review the different mechanical aspects at the origin of acceleration of organic transformations in water.

### (a) The hydrophobic effect

The hydrophobic effect plays an important role in many processes, including protein folding, substrate–enzyme binding, and micelle and bilayer formation; its origin at the fundamental level has been a topic of intense research and debate for many years.

**Fig. 4:**
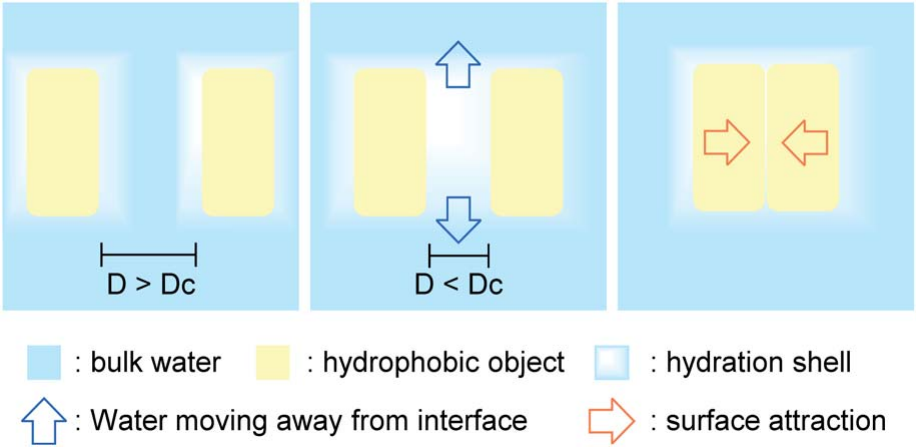
Hydrophobic effect leading to the merging of two hydrophobic entities.

When two large hydrophobic objects in high local concentration are close to each other, separated by a thin layer of water (thinner than the nanometer-scale critical distance, *D*_c_), the hydrogen-bond deficiency for the merged hydration shells induces a drying effect,^11,12^ causing water to migrate from this energetically unfavored state to the bulk water. The unbalanced pressure created by this vacant area causes the hydrophobic entities to converge (Fig. 4). The energy of the aggregate is then lower than the energy of the separated starting materials, as the surface of contact with water is greatly reduced. This phenomenon can be seen as an internal cohesive pressure effect.

By altering the nature of starting materials participating in Diels–Alder cycloadditions, Engberts *et al.* postulated that the hydrophobic effect was more pronounced due to hydrophobic interactions occurring closer to the reaction center.^13^ They also claimed that the hydrophobic effect on rate acceleration is not due to interfacial surface reduction, but to the loss of hydrophobic character near the activated complex. While the hydrophobic effect itself is important, it cannot solely account for the impressive rate accelerations observed. Otherwise, it would be comparable to running reactions under neat conditions.

While the strength of the interactions between water molecules (as opposed to interactions with the solute) is an intuitive physical explanation for hydrophobicity, the entropy cost to open a cavity in bulk water, due to its small size, can also be at the origin of this phenomenon.^14^

### (b) Cohesive energy density

Among solvents, water possesses one of the highest cohesive energy densities (550.2 cal mL^−1^). Cohesive energy density is defined as the amount of energy needed to completely remove a unit volume of molecules from their neighbors to infinite separation. Along these lines, a theory introduced by Lucas and Lee stipulates that it requires more energy to form a cavity for a reactant in water than in any other solvent, leading to a loss of entropy when a lipophilic substrate is added to water.^15,16^ Indeed, opening a cavity is entropically disfavored in any solvent, and this energetic loss is exacerbated due to the small size of water. This theory has only been validated by the “Mercedes-Benz” or MB model (a two-dimensional statistical mechanical model in which water molecules are represented as Lennard-Jones disks having Gaussian hydrogen-bonding arms),^17^ introduced by Silverstein in 1998, when small solutes are involved. Therefore, any reaction leading to a transition-state or product of smaller volume than that occupied by the reactants should be strongly accelerated, in order to occupy the smallest possible cavity. In the case of pathways leading to multiple isomers, the most compact transition state should be favored. This could have an impact on the stereoselectivity associated with, *e.g.*, Diels–Alder or Huisgen cycloaddition reactions, as the *endo* transition state occupies a smaller volume than the one leading to the *exo* product. This property is the direct consequence of the network of hydrogen-bonding between water molecules.

### (c) Hydrogen bonding

The addition of a non-polar molecule to water is characterized by a negative enthalpy Δ*H*, but a strongly positive overall free energy Δ*G* due to an unfavorable (*i.e.*, negative) entropic contribution, Δ*S* (eqn (1)).

1 Δ*G* = Δ*H* − *T*Δ*S*

**Fig. 5:**
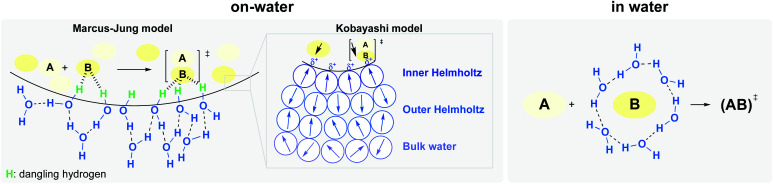
Hydrogen bonding under heterogeneous (left) and homogeneous (right) conditions, at the oil–water interface, and Kobayashi's partial polarization approach (center).

Two potential situations in water must be distinguished, involving either: (1) homogeneous (“in water”), or (2) heterogeneous (“on water”) conditions (Fig. 5).^18^ While the internal pressure reflects the cost of creating a cavity by reorientation of interfacial water molecules, the cohesive energy density is related to the cost of creating a cavity with complete disruption of water–water interactions. The former is the dominant parameter for small solutes (*vide supra*), while the latter becomes more important in the case of large solutes. Rearrangement of the water structure at the “oil”/H_2_O interface is illustrated in Fig. 5.

With small, dilute solutes, the aqueous interface is barely disturbed, as water molecules can reorganize themselves to avoid the loss of H-bonds towards the hydrophobic entity. In 1945, Frank and Evans explained the high entropic cost of adding a hydrophobic molecule to water by introducing the “iceberg model”. That is, around small, non-polar solutes, the first layers of water create a clathrate or hydrogen-bonded cluster to avoid “wasting” hydrogen bonds to the solute.^19^ Thus, the entropic cost can be explained by the “ordering” of water at the solute–water interface, and the enthalpy gain by the stronger bonds created around the solutes, compared to bulk water. This phenomenon has also been confirmed by the MB model.^17^ The iceberg case does not exist at higher temperatures, where the hydrophobic solvation is dominated by enthalpy. As the temperature increases, this “icy” shell structure disassembles before that of the bulk water structure. At a certain temperature, the sign of the transfer entropy Δ*S* becomes positive, as the strength and number of hydrogen bonds become predominant in bulk rather than at the interface. This behavior, by switching from entropy to enthalpy-driven, explains the high hydration heat capacity of water. To a smaller extent, multiple van der Waals water-solute and solute–solute interactions account for the enthalpy value.

In the case of large concentrated hydrophobic assemblies, leading to a heterogeneous system, the hydrogen-bonding compensation at the interface is geometrically impossible. Thus, a loss of hydrogen-bonding between adjacent molecules of H_2_O is observed. Sum-frequency generation spectroscopy (SFG)^20^ showed that the structure of water at the “oil”/water interface was characterized by the presence of free “dangling” hydroxyl groups accounting for ∼25% of the molecules at the aqueous interfacial layer. Those “dangling” OH-groups have been shown to protrude into the lipophilic area.^21–24^

Jung and Marcus also postulated that the explanation for the kinetic acceleration of “on water” Diels–Alder reactions lies at the boundary between the oil droplets and water, while the hydrophobic “bulk” behaves as a neat environment.^25^ The formation of hydrogen bonding between the dangling –OH and the lipophilic substrates plays a role in catalyzing reactions. Through DFT calculations derived from experimental rate constants, they showed that the activation energy is lowered by about 7 kcal mol^−1^ “on water” compared to neat conditions, if the transition state is “activated” by three H-bonds. Those hydrogen bonds are stronger in the transition state than towards the initial reactants. Based on their results, they postulated that the mechanism of the Diels–Alder reactions goes by a biradical intermediate under neat conditions, and by a concerted pathway in the presence of water. When water surrounds small hydrophobic solutes, the structure of the existing hydrogen bonds in the clathrate need to be broken to activate the substrates, thereby requiring more energy. Thus, as for large entities, a “H-bonding catalyst” effect is also postulated, but to a smaller extent due to this energy cost. This explains why the reaction is slower compared to its heterogeneous counterpart. The reaction is still accelerated because the energy required to break the interfacial H-bonding is lower than that in the bulk water.^26^

Additional proof that hydrogen-bonded water molecules orient themselves toward the hydrophobic layer (here made of CCl_4_ or hydrocarbons) has been provided by Richmond *et al.*, through vibrational studies.^27^ Kunieda *et al.* investigated the repartition of lipophilic mixtures in the presence of water.^28^ They showed that, while hydrocarbons were uniformly distributed in the oil phase, aromatic compounds were concentrated at the interfacial region. This phenomenon was attributed to weak hydrogen bonding between the aromatic rings and the water protons, which lowered the interfacial tension to a greater extent than with hydrocarbons. This study highlights the complexity of identifying a clear mechanism of action by water, due to the case-by-case nature of the partners involved.

Manna and Kumar studied the reaction between cyclopentadiene and alkyl acrylates and the impact of substrate concentration, either below their solubility limit (and therefore, remaining homogeneous), or above (and hence, heterogeneous).^29^ In dilute aqueous media, the activation enthalpy to the transition state is not affected by the reduction in hydrogen bonding capability of the acrylate. Under concentrated conditions (*i.e.*, “on water”), the activation enthalpy increased with decreased hydrogen bonding capability, indicating that less energy is being used to form hydrogen bonds. This suggests that hydrogen bonding is more involved in stabilizing the transition state in the case of heterogeneous conditions.

When hydrogen bonding plays a role as catalyst, favoring a particular transition state, then increasing the number of H-bonds by increasing the interfacial surface area should further enhance the reaction rate. Indeed, the same authors demonstrated, *via* optical measurements, the correlation between stirring speed and interfacial area of the reaction between cyclopentadiene and methyl acrylate in water. They demonstrated that the higher these values, the higher the rates of conversion.^29^

**Fig. 6:**
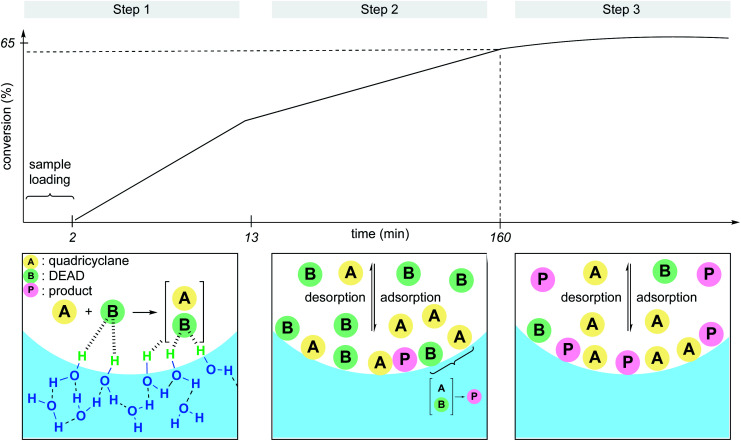
Interface adsorption/desorption in micro-droplets.

Whether the rate acceleration of the Diels–Alder reaction between quadricyclane and DEAD (diethyl azodicarboxylate) took its origin at the interface with the dangling –OH groups, or because of hydrodynamic effects (*e.g.*, vigorous stirring, ultrasonication) was unclear. To address this point, Zheng *et al.* developed a microfluidic device able to produce statically confined droplets in a glass capillary tube.^30^ Because DEAD is colored (orange-yellow), direct observation of the microdroplets containing the reactants could be performed *via* bright-field microscopy through the capillary tube in the absence of stirring. The conversion was also monitored by Raman spectroscopy from the intensity change at the characteristic peak of 1769 cm^−1^ for DEAD. Three distinct steps were identified throughout the sequence. In the first several minutes, the reaction begins, followed by a slower linear increase in conversion before finally leveling off at 65% conversion. The authors explained these observations as illustrated in Fig. 6. The slower rate in step 2 is due to an equilibrium between catalytic activation and the inhibition by adsorption and desorption of the molecules at the interface, although the majority of product (P) is formed under these conditions. In step 3, DEAD, being the limiting reagent, is present at insufficient quantities at the interface to be “activated” by the dangling –OH groups, in essence, shutting down product formation. They also noticed that smaller droplets gave rise to faster rates. While this report clearly reveals that activation at the interface is the predominant factor in the acceleration of this “on water” Diels–Alder reaction, it is important to consider the role of stirring to minimize the adsorption/desorption effect and surface saturation. Thus, steps 2 and 3 might not be observed under dynamic conditions.

Early work attempting to explain the role of water in accelerating “on water” reactions was all based on non-catalyzed reactions. Thus, literature was lacking explanations regarding the role of hydrogen bonding to lower the energy of catalyst–substrate-derived transition-states. Recently, Kitanosono and Kobayashi addressed this gap by considering partial polarization resulting from unbalanced H-bonding at the interface.^10^ They suggested a new “on water” model, where three layers of water, with different orientations, can be found at the interface (Fig. 5, center). This model aims to take catalysts into account, whether located in the aqueous or lipophilic phase. The first layer, where water molecules orient protons toward the hydrophobic phase (inner Helmholtz layer), is surrounded by a second layer (outer Helmholtz layer), and finally, by the bulk layer. The partial polarization of water at the interface would facilitate the formation of weak interactions with highly oriented transition states.

### (d) Solvent isotope effect

To further demonstrate the role of hydrogen bonds at the interface, the deuterium kinetic isotope effect has been studied by replacing H_2_O by D_2_O in “on water” reactions. While faster reaction rates are reported in H_2_O, although sometimes to a small extent, this phenomenon is not fully understood. Jung and Marcus highlighted the idea that physical factors, such as the higher viscosity of D_2_O, might affect shearing and could lead to bigger droplets resulting in smaller contact surface areas and thus, slower reaction rates. Grazziano demonstrated that, despite a larger cohesive density, D_2_O is a slightly better solvent for non-polar solutes than is its lighter counterpart. He explained that such results arise due to the formation of slightly larger cavities in heavy water.^31^

Beattie *et al.* first postulated that rapid “on water” reactions resulted from the protonation of the substrate S_1_ by water, stabilized by the resulting adsorbed hydroxide ion at the interface.^32^ Thus, the strong “on water effect” would be due to proton transfer, which, after reaction with the substrate S_2_, lead to the product P (eqn (2)).2
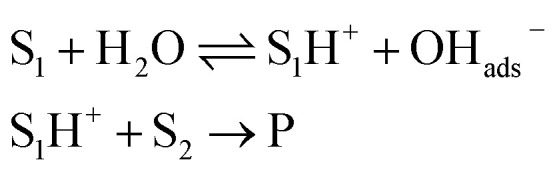


**Fig. 7:**
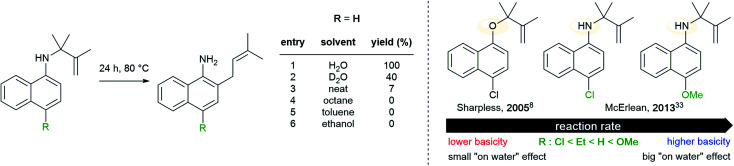
Correlation between “on water” effect and substrate basicity in an aza-Claisen rearrangement.

**Fig. 8:**
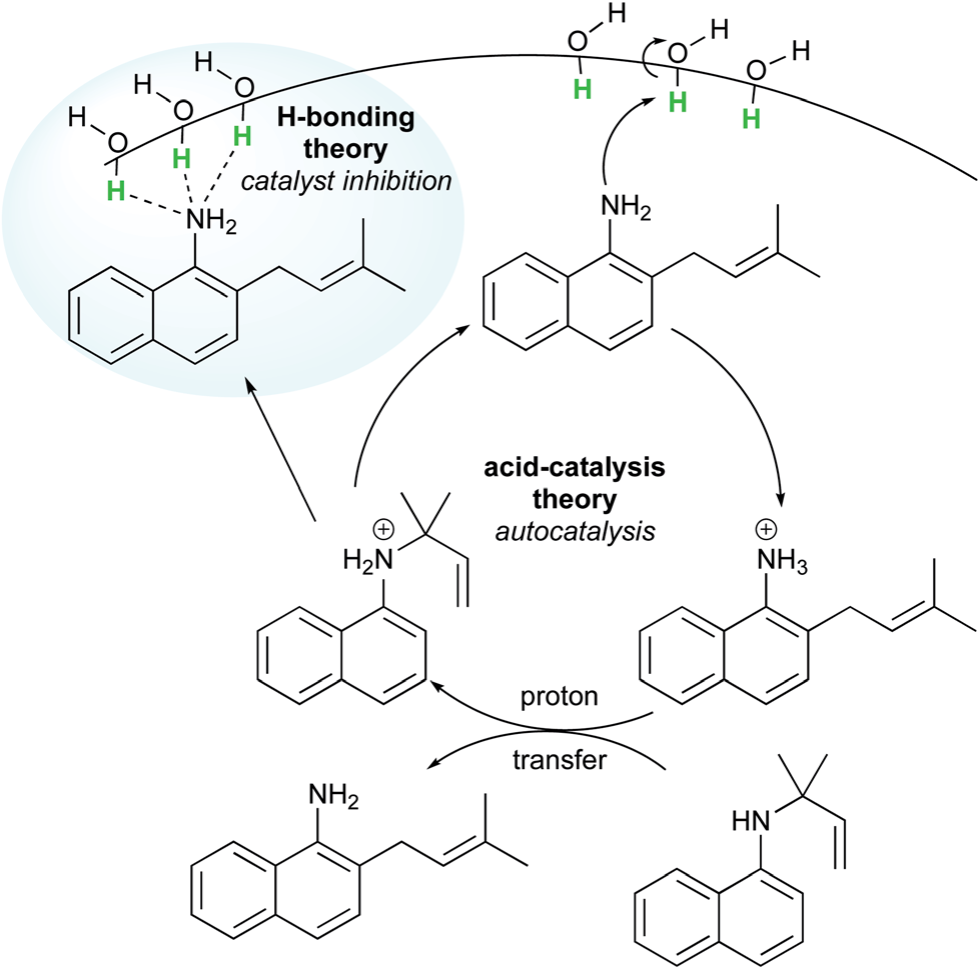
Autocatalytic aza-Claisen rearrangement facilitated by “on water” conditions.

Later, McErlean *et al.* suggested that the mode of activation by water was reactant-dependent. They postulated that, in the case of basic reactants, an acid–base mechanism at the water/organic interface was involved, leading to significant “on water” catalysis owing to a large *k*_H_/*k*_D_ isotope effect.^33^ When the substrate basicity is weaker, a hydrogen bond is responsible for substrate activation, leading to a small *k*_H_/*k*_D_ isotope effect and weak “on water” catalysis. This theory was illustrated by the aza-Claisen rearrangement, which usually requires high temperatures (200–300 °C) or acidic catalysis to render this process more practical. By contrast, total conversion could be achieved in water after 24 h at 80 °C, while the reaction failed in organic solvents or under neat conditions. The conversion in D_2_O only reached 40% over the same period of time. By changing the nature of the substituent R (in green in Fig. 7), the authors also noticed that the rate of reaction increased as did the basicity of the starting material. They also performed a direct comparison with the Claisen rearrangement described by Sharpless in 2005.^8^ With use of the less basic ether, the “on water” effect was only moderate (100% *vs.* 73% conversion neat). McErlean *et al.* also confirmed that, due to the higher basicity of the forming product over the starting material, an autocatalytic mechanism was involved (Fig. 8). Indeed, the rate plots show an induction period, followed by a rapid increase in rate. The involvement of the product in the catalysis was confirmed after the suppression of this induction period by doping the reaction with the final product, naphthylamine.

### (e) Soft and dispersed interface-rich aqueous systems

**Fig. 9:**
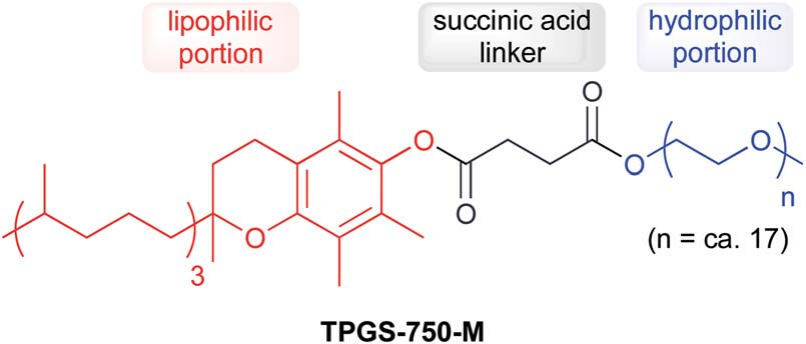
Structure of TPGS-750-M.

Due to water's unique properties, amphiphilic compounds often take on interesting organizational aspects in aqueous solution. When dissolved in water, amphiphiles such as surfactants self-aggregate into micelles wherein the hydrophilic head interacts with the aqueous phase and the hydrophobic tails collapse to form an inner section, commonly termed the “lipophilic core”, based on the hydrophobic effect. Researchers have leveraged these nanometer-sized particles as nanoreactors, housing organic substrates (due to their otherwise water-insolubility) leading to higher local substrate concentrations and hence, faster reaction rates.^34^ The designer surfactant TPGS-750-M (Fig. 9) has been found to display an apparently unique organizational arrangement of smaller micelles within a larger particle, providing sufficient lipophilicity to accommodate organic substrates and catalysts.^35^

**Fig. 10:**
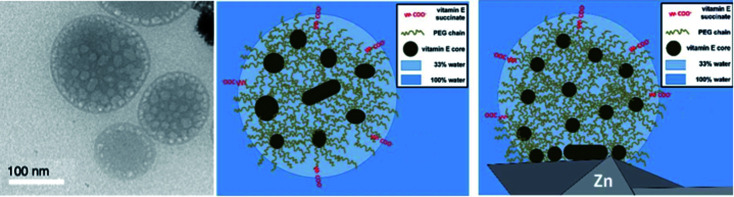
Observed *vs.* calculated arrangement for micelles derived from TPGS-750-M (left: cryo-TEM analysis; center: image based on calculations; right: image based on calculations in presence of zinc dust) (credit: Prof. Martin Andersson).

The average diameter of TPGS-750-M nanoparticles in aqueous solution was determined to be *ca.* 50 nm by dynamic light scattering (DLS) analysis; however, this size cannot be achieved by aligning surfactant molecules end-to-end; thus, nanoparticles observed *via* DLS cannot be individual micelles. DFT calculations performed by Andersson *et al.* suggested that these nanoparticles were comprised of 30–40 smaller micelles with diameters of 10–15 nm each, and with considerable amounts (estimated to be around 40% when using a co-solvent) of water in the PEG region.^36^ Cryo-TEM images support the existence of these micellar aggregates, also shown to exist in the presence of either 15 v/v% co-solvent, or triethylamine (Fig. 10). Because the individual micelles are within such close proximity to one another inside each aggregate, substrates and catalysts (as well as the products formed) can readily exchange through the surrounding water between them, thus accounting for the high efficiency observed under standard micellar catalysis conditions. The study also suggested that the micelle structures were stabilized by varying amounts of residual (0.1–10%) impurities (*e.g.*, vitamin E succinate), left behind from the synthesis of this surfactant, thereby reducing surface tension between the phases.

Interestingly, these calculations also indicated that zinc dust added to micellar solutions interacts with the lipophilic inner cores of the micelles and protects moisture-sensitive organozinc species, generated *in situ*, from water (Fig. 10, right). This explains why water-sensitive Negishi-like couplings are possible in aqueous surfactant solutions.^37^

**Fig. 11:**
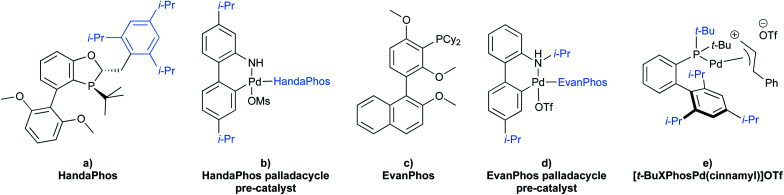
Structure of lipophilic ligands and pre-catalysts for micellar catalysis.

To gain maximum entry and residence within the hydrophobic inner cores of these nanoreactors, a ligand should possess high lipophilic character, and thus a higher log *P* value (calculated *n-*octanol/water partition coefficient) in aqueous micellar media. The design of HandaPhos relied on a branched and lipophilic triisopropylbenzyl moiety affixed to the oxaphosphine portion of the BI-DIME biaryl array (Fig. 11a). Ligand lipophilicity, electronic structure, and associated steric effects work synergistically leading to lower levels of required chelated Pd; only 1000 ppm (0.1 mol%) of this 1 : 1 complex is needed to catalyze Suzuki–Miyaura couplings.^38^ Incorporating isopropyl groups on ligands has been found, with some generality, to have a positive impact on the overall effectiveness of the derived transition metal-based catalyst. Hence, following the HandaPhos model, inclusion of isopropyl residues resulting in more highly lipophilic biaryl-based palladacycle pre-catalysts led, remarkably, to a generalized procedure in recyclable water (containing 2 weight percent TPGS-750-M) for Suzuki–Miyaura couplings^39^ at 300 ppm (0.03 mol%) Pd or lower (Fig. 11b). In addition, a more readily prepared ligand, EvanPhos (Fig. 11c), as part of a new palladacycle that, yet again, included isopropyl group substitutions at both carbon and nitrogen (Fig. 11d) was also developed for Suzuki–Miyaura, Heck, and Sonogashira couplings that involve 1500–3000 ppm (0.15–0.30 mol%) levels of this Pd catalyst.^40^

Beyond the hydrophobic effect, another feature worthy of note regarding the newly inserted isopropyl groups was also observed in the case of the same palladacycle. The *N*-isopropylcarbazole, formed from *in situ* decomposition of the palladacycle, competes less effectively for ligation of palladium. Direct comparison between *N-*isopropyl, *N*-methyl, and N–H carbazoles showed that the presence of the *N-*isopropyl group had little impact on the level of conversion, while both the *N*-methyl and *N*–H carbazoles clearly inhibit the coupling reaction to a greater extent (Table 4).^40^

**Table 4:** Effect of carbazole substituents on palladacycle-catalyzed Suzuki–Miyaura cross-coupling reactions

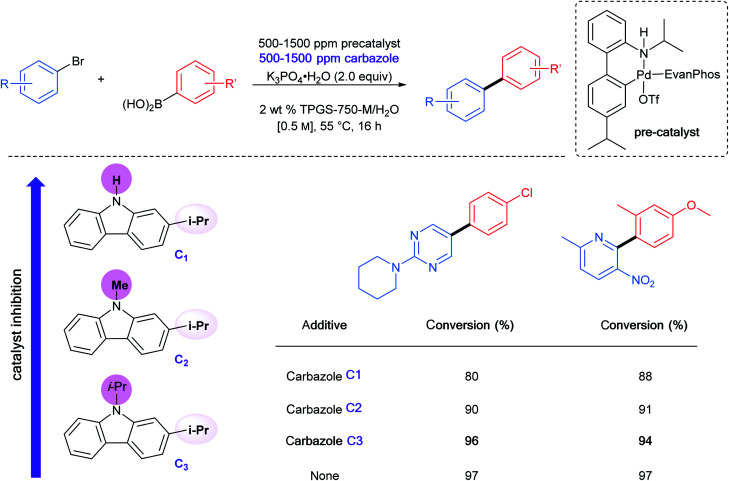

Similar aqueous micellar conditions have been used to form C–N bonds *via* amination reactions where a π-allylpalladium precursor was found to be the most effective catalyst. The allylic moiety initially on palladium and bearing the more lipophilic substituent (*i.e.*, phenyl > methyl > hydrogen) led to the most successful coupling (Fig. 11e). This approach enabled the preparation of a variety of key intermediates associated with several active pharmaceutical ingredients (APIs) using pre-catalyst loadings as low as 1000 ppm of Pd.^41^

**Fig. 12:**
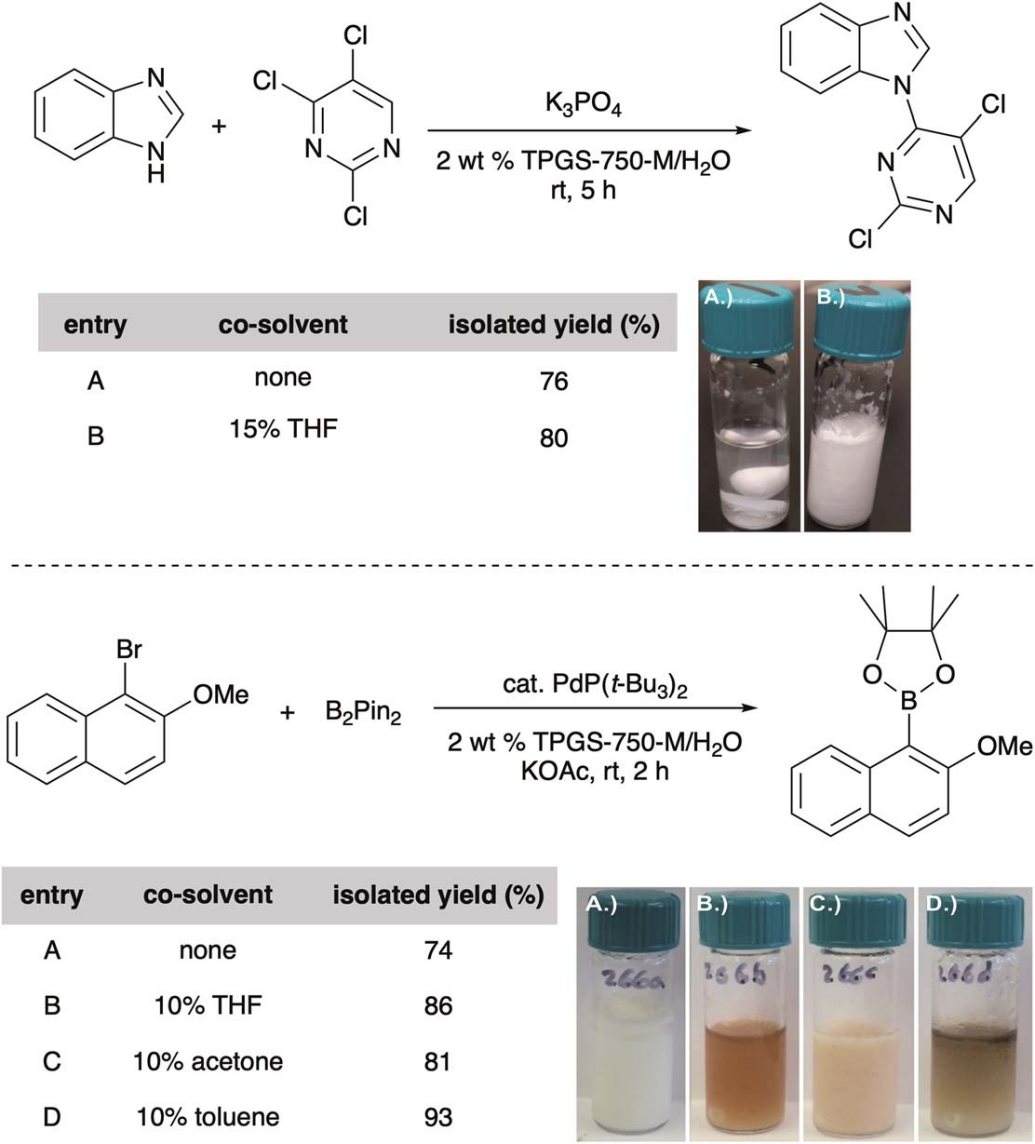
Effect of co-solvents on yields and processability.

Unlike reactions “on water” (*i.e.*, reactions run in the complete absence of any organic solvent), those carried out in micellar media tend to be responsive to the presence of co-solvents, typically used in the 1–10 v/v% range, although greater amounts have, on occasion, proven to be very effective as well.^42^ Here, solvents such as acetone, THF, PEG-200, and even EtOAc and toluene, have proven useful especially when one (or both) reaction partner is a water-insoluble solid. The co-solvent “trick” plays multiple roles, including (1) softening or, depending upon the amount used, dissolving the solute, aiding in its accessibility to the micellar cores, and thereby, usually increasing reaction rates; (2) expanding the micellar inner core size and thus, volume for accommodating substrates and catalyst by occupation; (3) enhancing the nature of the emulsion, in many cases ensuring good stirring of the reaction mixture (preventing agglomeration). The choice of a co-solvent is substrate(s) dependent, and a few are usually tested to achieve the desired reaction mixture properties (Fig. 12).

### (f) Lower metal loadings + milder reaction temperatures: the nano-to-nano effect

The “nano-to-nano” effect is one among several “new rules”^43^ that are at play when dealing with chemistry where water is the sole “solvent”, typically constituting 98% of the reaction medium by weight. It is the key element behind successful heterogeneous catalysis used under uncharacteristically mild micellar catalysis conditions. Among the many benefits of this phenomenon includes, importantly, usually ppm loadings of catalyst, whether involving precious or base transition metals. These unusual conditions result from two key factors working in harmony: (1) higher local concentrations of reactants found within nanomicelles; and (2) the hydrophilic (M)PEG chains of the surfactant serving as ligands for the nanoparticles (*e.g.*, of Pd, Ni, Cu), in effect delivering substrate-rich nanomicelles to the metal catalyst. The proximity of the catalyst to the reactants thus facilitates reaction, leading to the reduction of thermal energy input and hence, milder conditions.^44^

**Fig. 13:**
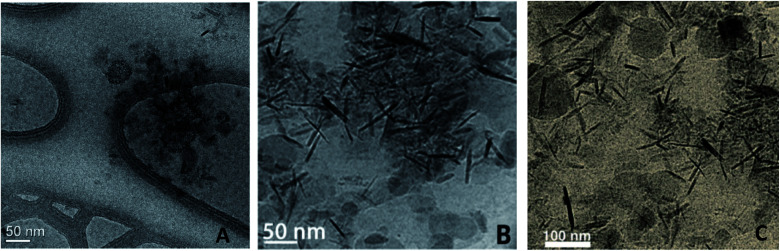
Cryo-TEM image of the “nano-to-nano” effect in reactions using nanomicelles together with metal-containing nanoparticles in (A) Cu-catalyzed click reactions; (B) Pd-catalyzed Suzuki–Miyaura and; (C) Sonogashira reactions.

The “nano-to-nano” effect relates to the *nano*micelles that house and deliver the educts to the *nano*particles of catalyst, thereby eliminating the typical need for applying heat as a means of increasing collisions between the substrate and catalyst. This delivery mechanism occurs due to the added stability that the metal catalyst receives *via* chelation by PEG oxygens acting as ligands (*i.e.*, stabilizing the metal which is looking to achieve 18 electron status). Such a phenomenon is easily observed *via* cryo-TEM analysis. The nanoparticles developed for Lindlar reductions,^45^ click reactions,^46^ Suzuki–Miyaura couplings,^47^ Sonogashira reactions,^48^ and nitro group reductions^49^ all lead to similar findings: the needle-like or rod-like shapes of the metal nanoparticles are associated with the TPGS-750-M surfactant-derived spherical nanomicelles (Fig. 13). This delivery mechanism, only operative in water, allows for reactions to be conducted using nanoparticle catalysts containing ppm levels of metal, including Pd (≤0.1 mol%).

### (g) Water sculpting effect on nanoparticles

**Fig. 14:**
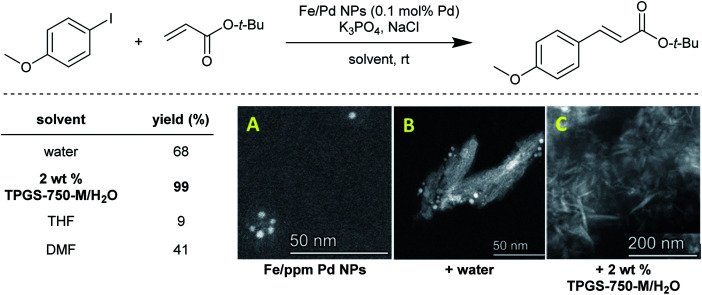
Solvent screening for Heck reaction, and STEM images of the evolution of Fe/Pd NPs in aqueous surfactant solution ((A) to (B) then to active catalyst (C)).

Typically, water is viewed as the medium in which “normal” micellar catalysis takes place (as opposed to inverted micellar catalysis, where the hydrophilic portion of each surfactant, in organic solvent, self-aggregates to form inner micellar cores, while the lipophilic sections occupy the outer area of each micelle). The substrates and catalysts are simply added and by thorough mixing, they gain entrance to the “solvent” inside the lipophilic cores, or the interfacial area between these and the surrounding water. But while the new rules for doing chemistry in water are very much still being discovered, there is yet another phenomenon that has been recently observed, referred to as the “water sculpting” effect. As shown for several Pd-catalyzed coupling reactions (*e.g.*, Sonogashira^48^ or Suzuki–Miyaura^47^), Fe/ppm Pd nanoparticles (NPs) that are initially prepared in THF and are spherical in nature have their shape and size (*ca.* 1–5 nm) “sculpted” into far larger nanorods (*ca.* 100 nm) simply upon exposure to water (Fig. 14). This medium acts by dissolving significant levels of the Mg and Cl ions present in the original makeup of these NPs. Moreover, the crucial ligand contained within each set of NPs (*e.g.*, SPhos, XPhos) is also released into the aqueous medium, virtually eliminating catalyst activity if the aqueous medium is removed and replaced by fresh water. In the presence of nanomicelles, however, the phosphine is presumably accommodated within the micellar cores, and is available for the newly formed nanorods that together, function very effectively as catalysts. Attempts to use these same NPs (as originally formed) in organic solvents (*e.g.*, THF and DMF), rather than in aqueous mixtures, led to no change in the original shape of the NPs and not surprisingly, limited catalysis. Clearly, the water functions to both convert the initial NPs into active catalysts, and then as the reaction medium for that catalysis.^50^ At this stage, the challenges associated with characterization of the reactive systems and the reliable generation of the true active species from the pre-catalysts remain a significant impediment to the widespread and on-scale implementation of these heterogeneous reagents.

### (h) Enzymatic catalysis in the presence of micelles: the “reservoir” effect

The use of both naturally occurring enzymes, as well as those created *via* directed evolution, are attractive tools in the chemist's toolbox, and have gained even further attention owing to the Nobel Prize awarded in this area in 2018.^51^ The selectivity of their bio-catalysis, if not specificity, is oftentimes difficult to match using chemo-catalysis, along with the typically mild reaction conditions, noteworthy safety profile, and of course, their use in buffered aqueous media. Their alternative use in organic solvents applied to synthetic problems dates back to the last century, rationalized on the basis of (1) high solubility of most organic compounds in nonaqueous media; (2) relatively quick product recovery from organic solvents compared to water; and (3) insolubility of enzymes in organic media that allows for their recovery and reuse (Table 5).^52^ Two approaches to enzymatic catalysis were attempted in organic media: the first is to directly suspend the lyophilized enzyme powder in organic solvents; the second is to apply an aqueous protein solution to the surfactant-containing organic solvent, thereby forming “reverse micelles”.^53,54^

**Table 5:** Comparison of enzymatic reactions in organic solvents, aqueous buffer and micellar aqueous buffer

Medium	Organic solvents	Aqueous buffer	Micellar aqueous buffer
Solubility of organic materials	Excellent	Poor	Good
Solubility of enzymes	Poor	Excellent	Excellent
Enzyme conformational mobility	Poor	Excellent	Excellent
Control of pH	Poor	Good	Good
Enzymatic inhibition by organic materials	Yes	Yes	Reduced

Water, however, is irreplaceable as the medium for most enzymatic catalysis; its high dielectric constant and hydrogen bonding properties continue to play major roles, as they have throughout evolution. Moreover, in organic solvents, enzymes may be denatured and lose their conformational stability and native structure, in addition to their lack of solubility in the absence of water.^55^ And with respect to pH, an especially influential parameter, this has no meaning in organic solvents.^56^ Enzymatic catalysis, as utilized in water and as applied to synthetic chemistry, is not without its share of obstacles. For example, not only might the initial organic substrate have solubility issues, but perhaps more importantly, entrance to the active site may end up being blocked as the water-insoluble product accumulates, leading to textbook enzymatic inhibition.^57^ This phenomenon is oftentimes substrate and especially product-dependent, and can dramatically decrease the extent of conversion for a given transformation. Fortunately, there may be a very simple experimental “fix” to this problem that avoids the otherwise common reliance on varying percentages of DMSO, which as a dipolar aprotic solvent in and of itself, from the environmental perspective, is especially egregious.^58^ Recently, it has been observed, for example, that the reactivity of ketoreductase ADH101 towards (*E*)-4-phenyl-3-buten-2-one reached a plateau at 57% conversion after one hour in a buffered aqueous medium (Table 6, entry 1). However, simply adding 2 weight% of any one of several common surfactants to the buffer (*e.g.*, Tween 60, Triton X-100, TPGS-750-M), was found to improve both the rate and level of conversion for these enzyme-catalyzed ketone reductions. Most notably, the conversion increased with increasing surfactant concentration (entries 2–4). These observations suggest that the nanomicelles present in the aqueous reaction medium serve as a reservoir for both educts and products, regulating their concentrations by providing alternative “housing” to the enzymatic pocket (Table 5). This dynamic exchange, *i.e.*, where products are drawn away from the enzymatic pocket and to the lipophilic micellar interior, such that water-insoluble substrates can gain access, lead to what can be significantly improved levels of conversion and hence, isolated chemical yields.^59,60^

**Table 6:** Higher surfactant concentration increases reaction conversion

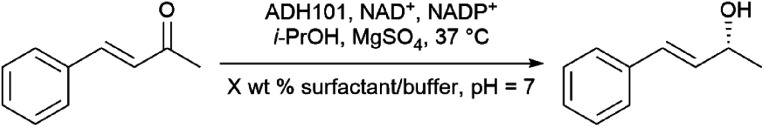		
Entry	TPGS-750-M (wt%)	Conversion at t = 1 h (%)
1	0	57
2	2	67
3	4	72
4	6	75

### (i) Accommodating polar organic molecules: MC-1 and PS-750-M

**Fig. 15:**
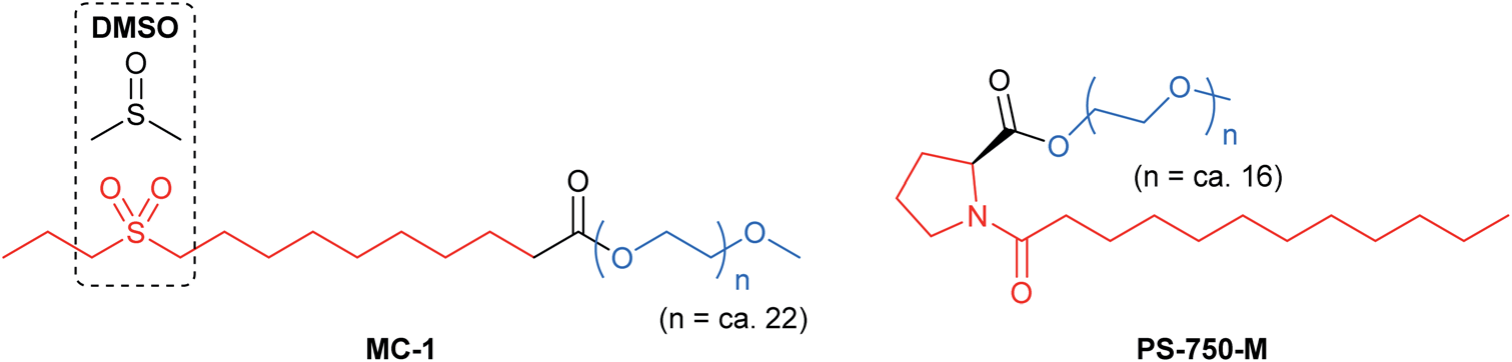
Structures of MC-1 and PS-750-M.

Efforts remain underway to continuously develop new designer surfactants that accommodate either a wider range of substrates, or a specific reaction type. For example, MC-1 was developed to address the solubility issue associated with peptide bond constructions in water that involve polar amino acid-containing substrates. Its preparation was inspired by the commonly used organic solvent for related peptide couplings, DMSO (Fig. 15, left). The sulfone group was embedded within the lipophilic tail such that when MC-1 is dissolved in water, it forms micelles containing more “polar” (typically) non-polar inner cores that accommodate amino acid/peptide partners.^61^ Similarly, PS-750-M^62^ (Fig. 15, right) imitates amide solvents (*e.g.*, DMF and NMP), accommodating substrates with moderate-to-high polarities, *e.g.*, nitroalkanes^63^ and hydrazones.^64^ Hence, this leads to better stirring, higher conversions, and thus, improved yields. Most importantly, from the green chemistry perspective, this technology enables reactions to take place in water under ambient conditions involving polar compounds to the exclusion of toxic dipolar aprotic organic solvents.^65^

### (j) Going up in foam? Try Coolade

**Fig. 16:**
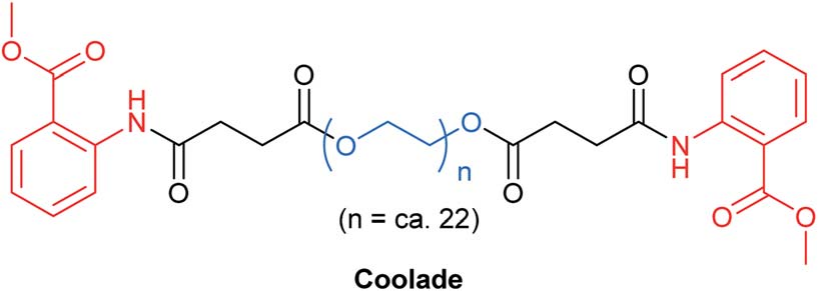
Structure of Coolade.

As with most surfactants, designer surfactants (*e.g.*, TPGS-750-M,^35^ Nok,^66^ PTS,^67^ PS-750-M^62^) have a propensity to foam, from either reactions that require gases or those which generate gas. This phenomenon can be problematic, requiring special attention and planning, such as providing extra headspace above a reaction mixture. Pioneering work by Tamura *et al.* has shown that foamability of aqueous surfactant solutions linearly decreases with *decreasing* length of the hydrocarbon chain emanating from the polar head group.^68^ This led to the creation of Coolade, a designer surfactant formulated without a hydrocarbon tail.^69^ Methyl anthranilate, a flavoring agent with a grape scent, was added to both ends of a hydrophilic PEG chain bearing succinic acid ester linkages (Fig. 16). Upon dissolution in water, it self-aggregates into nanomicelles. As expected, under normal conditions of use, aqueous solutions of Coolade do not foam. This key characteristic has been used to great advantage for nitro group reductions (using NaBH_4_ + Fe/ppm Pd NPs),^49^ and azide reductions (with Zn + NH_4_Cl).^69^ Moreover, when designing the route to Coolade, green chemistry principles were fully considered.

### (k) Cloud point temperature for surfactants

Much of the above discussion is focused on nonionic surfactant-based micelles functioning as nanoreactors. A common mistake that can disassemble or lead to the coalescence of micelles is the application of too much heat to a micellar solution. This varies according to the surfactant, especially when the temperature is above its cloud point. At elevated temperatures, a nonionic surfactant can undergo phase separation (into a surfactant-rich phase and a surfactant-poor phase), turning the solution cloudy.^70^ The resulting properties of such mixtures can be very different from those of the original aqueous surfactant solution. Due to the rise in temperature, the ethoxylate chains (within the PEG portion) lose water, becoming less coiled and more hydrophobic and hence, cloudy. The shape of the micellar array may also reorganize to more highly aggregated structures (*e.g.*, from spheres into vesicles), thereby altering the reaction medium.^71^ Therefore, micellar catalysis is typically done under relatively mild conditions (*i.e.*, below its cloud point).

### (l) Polymeric cellulose as an alternative to surfactants

**Fig. 17:**
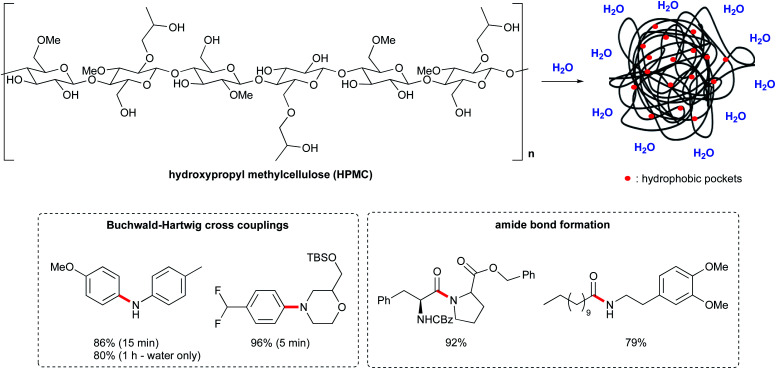
Hydroxypropyl methylcellulose (HPMC) structure and applications.

Recently, a new approach to organic synthesis in water has been reported by Abbvie.^72^ Instead of using surfactant-based micelles, this new technology relies on a polymer matrix, hydroxypropyl methyl cellulose (HPMC), which is a benign food additive commonly used as a thickening agent. It is also employed in drug delivery, to control the release of both hydrophilic and lipophilic APIs. The addition of HPMC to water (0.1–2 wt%) leads to remarkable rate enhancements, with reaction times on the order of minutes, and in some cases, even seconds.^73^ The homogeneity of the reaction mixture was also greatly improved, which is important for scale up processes. The mechanism is unclear but the presence of small hydrophobic pockets within a polymer matrix might favor the reaction, *via* the hydrophobic effect (Fig. 17). Indeed, lipophilic substrates seem to benefit from this environment. Additionally, the free hydroxyl groups from cellulose could act as hydrogen donors. While it is used as an emulsifier in formulation, it does not form micelles in water.

Given these advances focusing on chemistry in water, it is not surprising that the number of applications has begun to grow, infiltrating most types of reactions in both the chemo- and bio-catalysis regimes.

## 4. Applications

Chemistry in water as the sole “solvent” now includes numerous types of organic transformations. The simplicity of work-up, which is typically done *via* “in flask” extraction of the product from the aqueous reaction mixture using a single, minimal amount of a recyclable organic solvent, or by simply decanting or filtering to obtain the solid product, makes water an attractive medium. In addition to the economic and environmental advantages, water can also have a dramatic positive effect on reactivity and selectivity. Below are selected, representative examples, with an emphasis on mechanistic considerations.

### (a) Cycloadditions

**Fig. 18:**
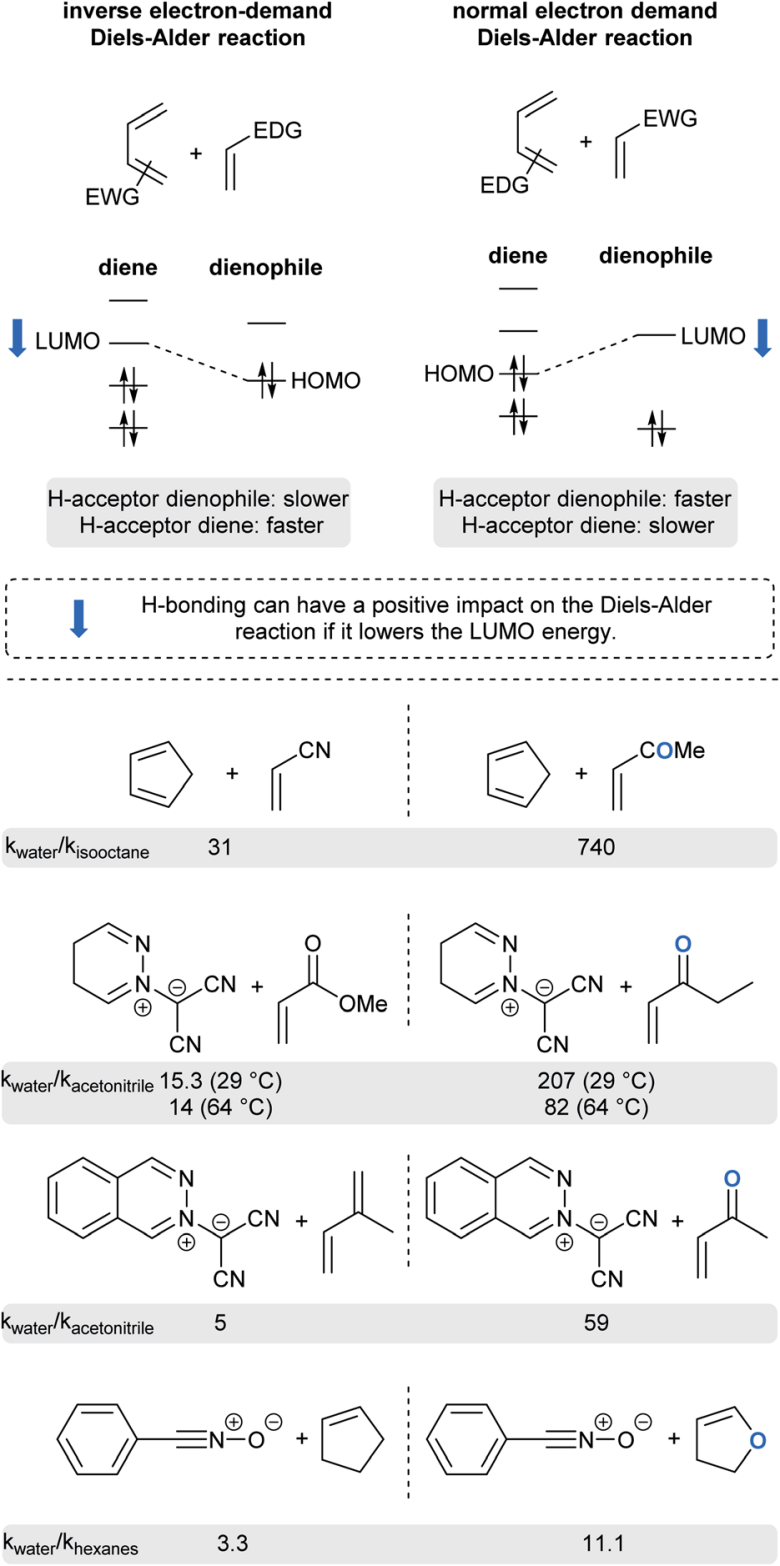
Impact of H-bonding on the frontier orbital energies in pericyclic reactions.

Whereas rates of cycloadditions tend to be relatively unaffected by the choice of organic solvent, the hydrophobic effect in aqueous media can lead to significant rate enhancements. Due to hydrogen bonding or acidic catalysis with substrates, dangling –OH groups at the “oil”/H_2_O interface can influence reactivity *via* substrate activation and transition state stabilization, but only when H-bond acceptors are present in one or both substrates. Such hydrogen bonding withdraws electron density, thereby lowering the energy of the frontier orbitals. This could be beneficial, or detrimental, depending on which orbital of which substrate has its energy lowered.^74^ For normal-demand Diels–Alder cycloadditions, lowering the energy of the LUMO of the dienophile reduces the energy gap in the transition state, whereas reducing the energy of the HOMO of the diene has the opposite effect. For inverse-demand Diels–Alder cycloadditions, lowering the LUMO of the diene reduces the gap, while reducing the HOMO of the dienophile disfavors the reaction. This is nicely illustrated by the ratio of reaction rates for pericyclic reactions in water *vs.* organic solvents for selected examples (Fig. 18).^5,75–77^ Thus, when a ketone is present in the dienophile or dipolarophile, the reaction is significantly accelerated. An ester, being a weaker H-bond acceptor, has a correspondingly reduced influence. Finally, molecules with only a soft H-bond acceptor (such as isoprene or cyclopentene) showed only a small rate acceleration due to the hydrophobic effect.

Interestingly, reactions of methyl acrylate and ethyl vinyl ketone with pyridazinium dicyanomethanide demonstrated different behavior in water compared to acetonitrile. With the ketone, the relative rate acceleration in water was 207 at 29 °C but dropped to 82 as the temperature increased to 64 °C. With the analogous ester, the relative rate acceleration was only 15, but remained steady with increasing temperature.^76^ Butler *et al.* hypothesized that water molecules structurally organized around the transition state to form hydrogen bonds with the ethyl vinyl ketone (ketones being water “super” dipolarophiles). On the other hand, water “normal” dipolarophiles, such as esters, do not form comparatively strong hydrogen bonds. Thus, the observed moderate rate acceleration can only be attributed to the hydrophobic effect. Increasing the reaction temperature disrupts H-bonding, thereby affecting solely the case of the ketone.

In order to assess the relative contributions to reactions involving hydrophobic and/or hydrogen-bonding effects, computational studies were carried out by Furlani and Gao.^78^ Diels–Alder cycloadditions between cyclopentadiene and either methyl vinyl ketone (MVK; capable of accepting H-bonds), or isoprene (not capable of accepting H-bonds), were investigated in aqueous media. Their data suggest that the impact of the hydrophobic effect depends on the nature of both reactants. They showed that the overall free energy continuously decreases as cyclopentadiene and isoprene get closer, perhaps due to a reduction in hydrophobic surface area. On the other hand, the free energy associated with the reaction involving MVK fluctuated as the number of hydrogen bonds varied with the distance between reactants. They concluded that, despite a rough estimation of the H-bonding contribution, both effects contribute almost equally to stabilize the transition state with MVK, while the hydrophobic effect alone (−4.6 kcal mol^−1^) plays a role with isoprene.

**Fig. 19:**
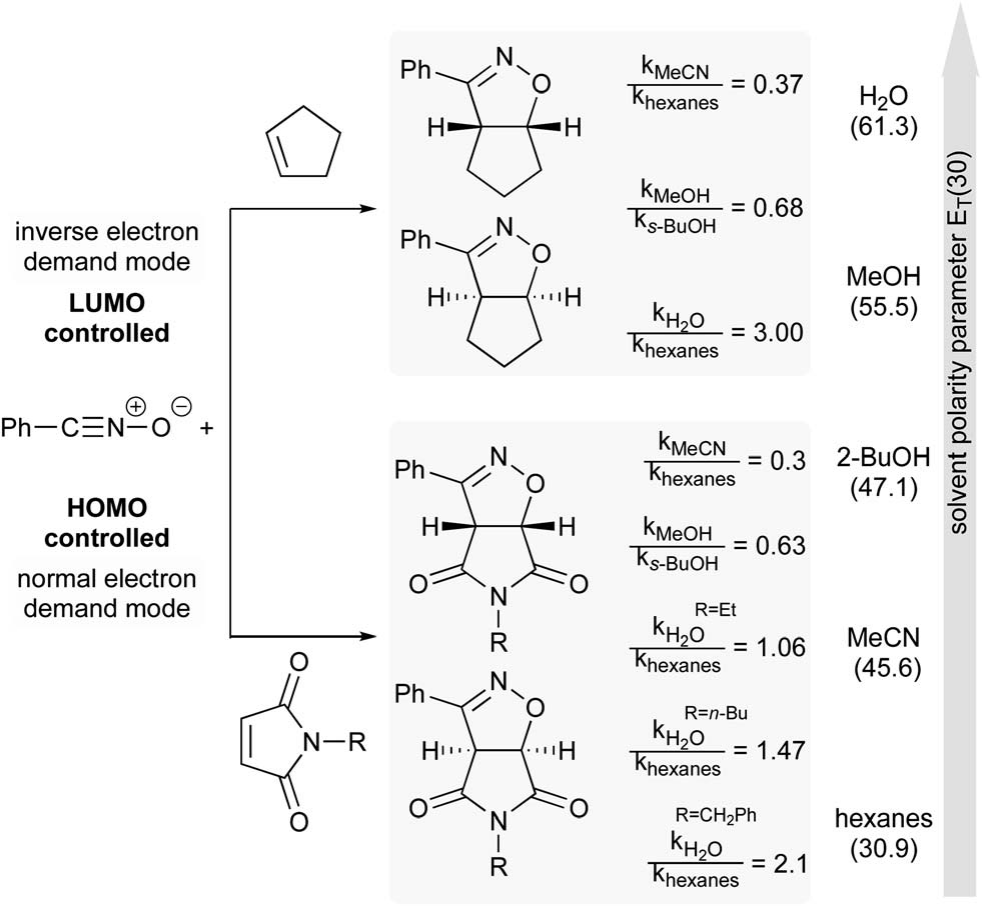
Influence of solvent polarity on reaction rate.^74^

The next example illustrates the complex interdependence of these three parameters (polarity, H-bonding and hydrophobic effect), and how they can impact the rate acceleration of 1,3-dipolar cycloadditions between, *e.g.*, cyclopentene or *N*-substituted maleimide and benzonitrile oxide (Fig. 19).^77,79^ Initial consideration was given to the polarity of the solvent. The relative rate constant is lower in more polar solvents, except for water, in which reactions were the most rapid. The importance of H-bonding was also brought to light by changing the nature of the dipolarophile. For *N*-substituted-maleimides, although both reaction partners are capable of hydrogen bonding, it might be postulated that the frontier molecular orbital of benzonitrile oxide is more affected than that of the maleimide, thus leading to reduced reaction rates (*cf.*Fig. 18). Here, both polarity and H-bond donating ability play a role, but in opposite directions, whereas they work in harmony for most Diels–Alder reactions. Finally, the impact of hydrophobicity is readily seen by varying the nature of the substituent on nitrogen in the maleimide. Increasing its lipophilicity increases the relative rate constant in water. Hydrophobic interactions lower the Gibbs energy of activation by generating a less hydrophobic-activated transition state relative to individually hydrated hydrophobic molecules.

“On water” conditions are especially useful for reactions characterized by a negative volume of activation. Indeed, there is an energetic advantage to reducing both the interfacial area with water and occupying the smallest possible cavity, which is the case when the molar volume of the transition state is smaller than that of the separated starting materials. In that regard, not only did Breslow *et al.* observe a rate acceleration in the cycloaddition between cyclopentadiene and butanone when the reaction was performed “on water” *vs.* neat, but they also reported an enhancement of the *endo* : *exo* ratio (21.4 and 3.85, respectively) regardless of whether the reactions were carried out at high or low concentrations.^80,81^ In this example, only cyclopentadiene is insoluble in water. Another example, involving the more lipophilic dimethyl maleate, followed the same trend (*endo* : *exo* ratio = 13.7 *vs.* 2.8). They also noted that the use of an anionic, or cationic surfactant (SDS and CTAB) did not improve the product ratios.

Thus, the influence of water on the stereoselectivity of Diels–Alder reactions can be attributed to its high cohesive energy density, its preference for smaller transition-state volumes, the hydrophobic effect and, depending on the substrates, the hydrogen bonds that can be formed.

### (b) Aldol condensations

Another example of a reaction involving negative activation volume (−10 to −15 mL mol^−1^) is the Mukaiyama aldol reaction. An early report by Yamamoto and co-workers found that the *syn* product is favored under pressure,^82^ suggesting it has a smaller transition state than the *anti* isomer. In a similar vein, Lubineau observed that the *syn* isomer was favored in water in the reaction of a trimethylsilyl enol ether and benzaldehyde, while the same reaction catalyzed by TiCl_4_ in dichloromethane led to opposite stereoselectivity.^83^ While the yield was low, no reaction was observed in organic solvents in the absence of catalysts. These mild conditions allowed access to products, in the absence of any acid (*e.g.*, TiCl_4_) or base, which may also lead to the dehydration product (Table 7).^84^

**Table 7:** Reverse selectivity by switching to water in the Mukaiyama aldol reaction

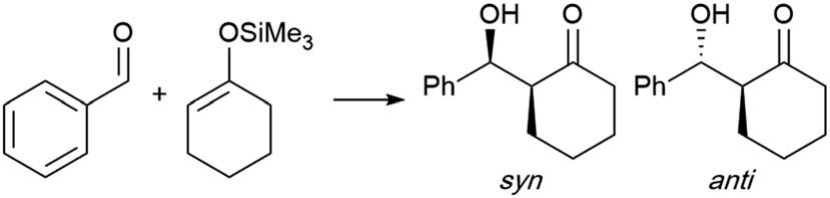				
Solvent	Pressure	Catalyst	Yield (%)	*syn* : *anti*
H_2_O	atm.	—	23	85 : 15
CH_2_Cl_2_	atm.	TiCl_4_	82	25 : 75
CH_2_Cl_2_	atm.	—	0	—
CH_2_Cl_2_	10 kbar	—	90	75 : 25
Toluene	atm.	—	0	—
THF	atm.	—	0	—
CH_3_CN	atm.	—	0	—

**Fig. 20:**
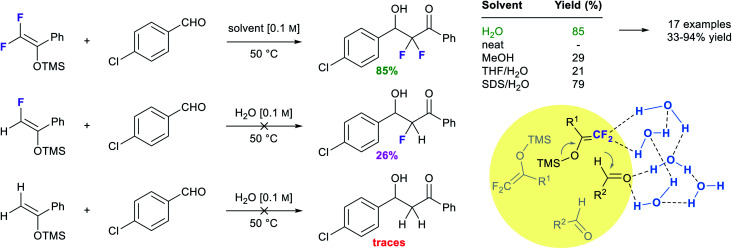
H-Bonding abilities of fluorinated substrates involved in Mukaiyama aldol reactions.

Zhou *et al.* took advantage of the hydrogen bonding network at the interface to “catalyze” the catalyst-free Mukaiyama-aldol reaction of difluoroenoxysilanes with carbonyl compounds (Fig. 20).^85^ The authors postulated that the C–F⋯H–O and the C=O⋯H–O interactions between the substrates and the dangling –OH groups from interfacial water allowed the two partners to arrange in a favorable orientation to one another, thereby promoting the reaction. While the reaction yield did not exceed 29% in organic solvents and failed under neat conditions, it reached 85% yield in water at 50 °C. Homogeneous conditions in the presence of THF led to poor results. In the case of micellar conditions in presence of SDS, the high local concentration and the hydrophobic effect might be responsible for the high yield (79%). When the –CF_2_ moiety was replaced by a –CHF or a –CH_2_, reducing the hydrogen bonding potential of the nucleophile, the reaction rate was greatly affected. DFT calculations supported the theory of cooperative interactions between the fluorides and five interfacial water molecules, lowering the activation barrier by 10.3 kcal mol^−1^.

### (c) Multi-component reactions

**Fig. 21:**
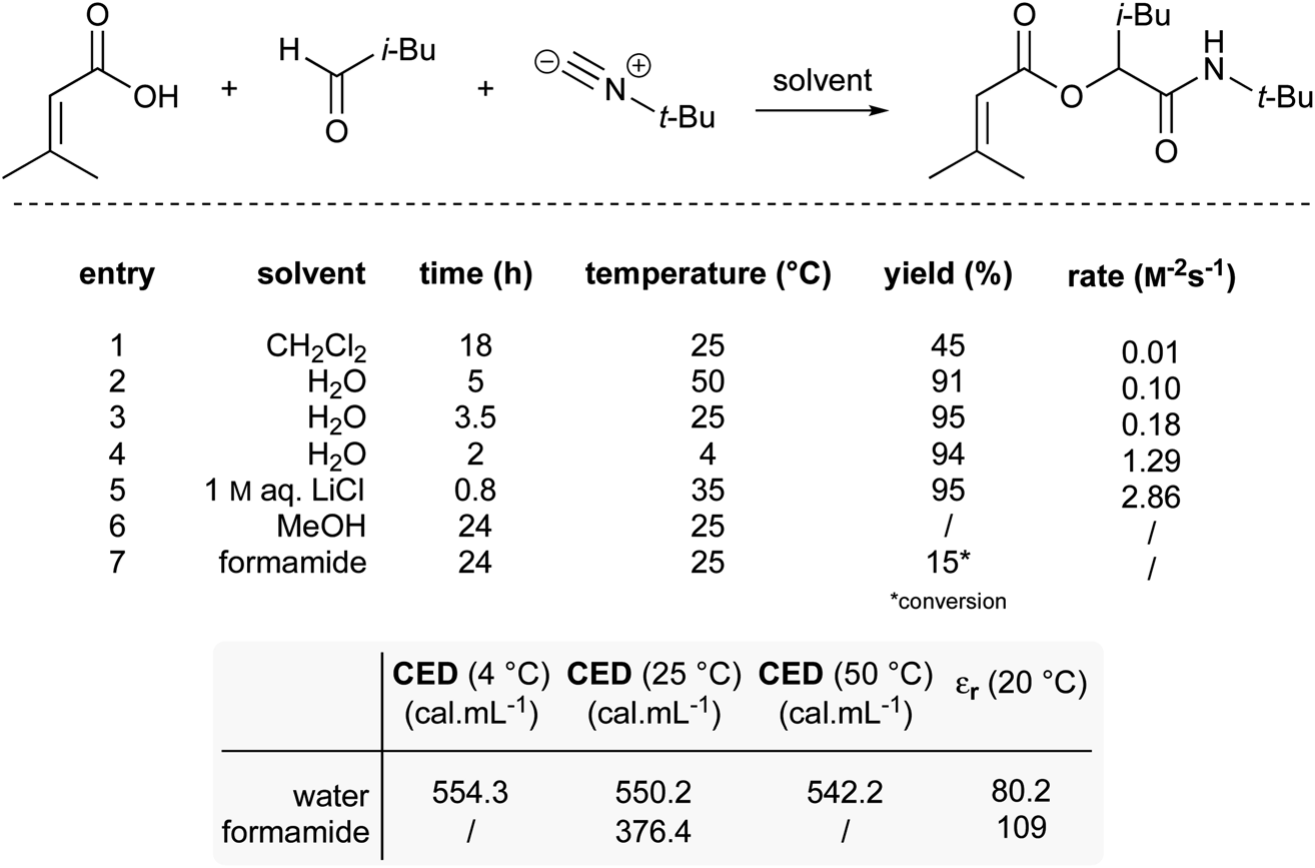
Effect of the high cohesive energy density of water on a Passerini reaction.

A third example that exhibits negative activation volumes includes multi-component reactions, due to the combination of multiple molecules forming a single intermediate and eventual product. This type of reaction can be accelerated using “on water” conditions due to the hydrophobic effect. The Passerini reaction, illustrated in Fig. 21, was performed in dichloromethane with modest yield after 18 hours, while it reached completion in water after only 3.5 hours.^86^ The absence of conversion in methanol (entry 6) indicates that the protic properties of water are not the key driving force. When formamide, characterized by a higher dielectric constant (109) but a lower cohesive energy density (CED) compared to water (dielectric constant = 80), was used as solvent (entry 7), only 15% conversion was observed. This result excludes the role that charge stabilization could play. Instead, this highlights the influence of the high cohesive energy density of water on reaction rate. As the cohesive energy density decreases with temperature, this would also explain why the reaction is faster at 4 °C (11% faster than at 25 °C; entry 4) but slower at elevated temperature (44% slower at 50 °C; entry 2). As previously reported,^87^ salting-out agents such as LiCl (entry 5) led to a 16-fold acceleration over pure water.

Additionally, the use of dioctadecyldimethylammonium bromide, a cationic surfactant forming micelles in water, has been reported to promote the Passerini multicomponent reaction with higher yields than those obtained in either pure water or dichloromethane.^88^ The nanoreactor cores host these intermolecular couplings, leading to α-acyloxy carboxamides with greater efficiency. Similarly, use of aqueous surfactants have also been shown to be useful for four-component Ugi reactions.^89^

### (d) Organometallic reactions

Many, if not most organometallics are highly polarized compounds that can also act as strong bases. Hence, they may be unstable in the presence of water, especially when the metal belongs to the s-block of elements. Consequently, textbooks recommend performing reactions involving organometallic reagents in organic solvents under strictly anhydrous conditions. Despite these pre-conceived teachings, several reports of such reactions of supposedly highly sensitive organometallics in water have now appeared. To prevent immediate quenching *via* protonation of the carbon–metal bond, different approaches have been taken, such as: (1) increasing the covalent character of the carbon–metal bond, by choosing a metal from groups 13–15,^90^ thus reducing its carbanion character and its sensitivity toward water; (2) designing a radical pathway, as radical intermediates are usually neutral and stable towards water; (3) leveraging compartmentalization to segregate the organometallic species from water with either “on water” or micellar catalysis conditions.^91^ The development of organoindium reagents is a noteworthy example of increased covalency leading to stable catalysts for aqueous applications^92,93^

**Fig. 22:**
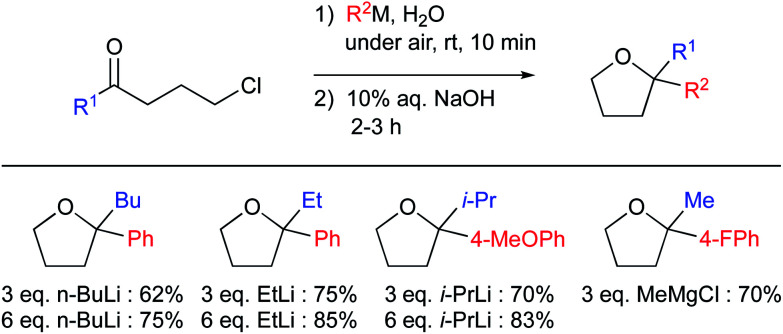
“On water” 1,2-additions of organolithium and Grignard reagents.

Recently, Capriati and co-workers reported both alkylation and arylation of aryl-γ-chloroketones affording good yields when run in water at room temperature (Fig. 22).^94^ They made use of “on water” conditions to perform the reaction competitively relative to undesired protonolysis. Identical reactions did not give satisfactory results in alcohols, confirming that the unique properties of water are crucial for success of these Grignard additions. That is, the dangling H-bond interactions (in this case, the authors postulated these as responsible for activation of the carbonyl derivatives), reagent hydrophobicity (probably providing shielding of the organometallic reagent within the hydrophobic phase, minimizing, or even preventing protonolysis), and likely self-cluster formations. They also reported a small solvent isotopic effect to account for the “on water” conditions.

**Fig. 23:**
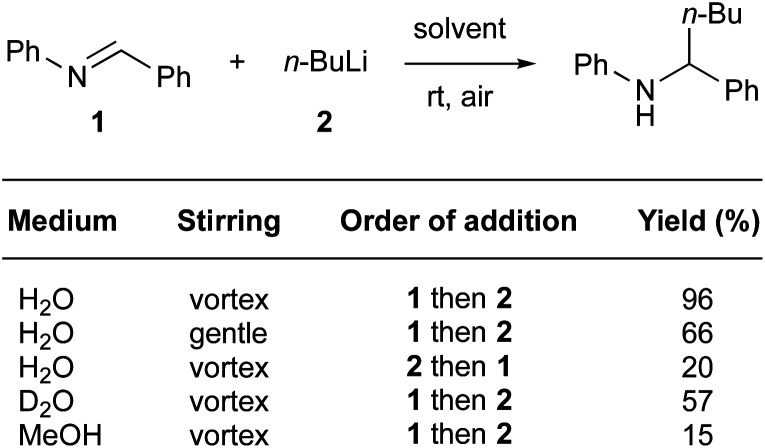
Nucleophilic additions of organolithium reagents to imines “on water”.

“On water” conditions have also been favorable for nucleophilic additions of organolithium reagents to imines (Fig. 23).^95^ Capriati *et al.* have highlighted the benefits of using water, as opposed to organic solvents, as the reaction medium. They mentioned that, due to the relatively poor electrophilicity of the imine in conventional solvents such as ether or hydrocarbons, the reaction hardly proceeds. While use of protic solvents such as methanol leads mainly to protonolysis, “on water” conditions tend to disfavor this side-reaction, probably due to strong intermolecular bonding between the surrounding water molecules. Thus, while the reaction in methanol led to poor conversion (15% yield), “on water” conditions afforded the desired product in a remarkable 96% yield (99% yield on a 5.5 mmol scale). Consistent with other reports,^96^ stirring is important to achieve high levels of conversion. Thus, fast stirring (vortexing) led to considerably higher conversion than did gentle stirring (96 *vs.* 66% yield). When *n*-BuLi was the first of the two reactants added to water, the yield dropped to 20%. It seems that the presence of lipophilic droplets is required to “shield” the organolithium from water, thus avoiding protonolysis. The reaction “on D_2_O” highlighted a significant isotope effect, as the yield dropped to 57%. These data are consistent with proton transfer or activation by interfacial water molecules.

### (e) Radical reactions

**Fig. 24:**
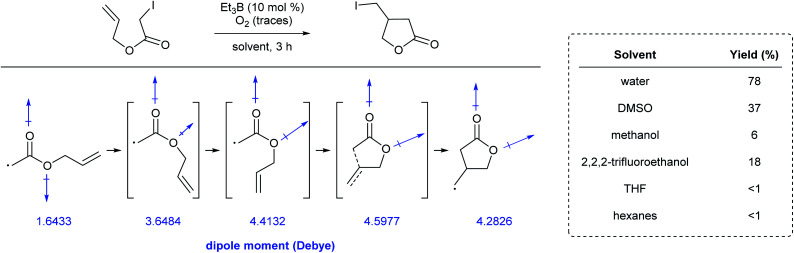
Dipole moment of rotomeric radical intermediates in cyclization reactions.

Yorimitsu *et al.* reported the synthesis of a range of lactones through atom-transfer radical cyclizations in various reaction media.^97^ Conversions were significantly higher in water than in benzene or hexanes. Since polar solvents stabilize molecules bearing a large internal dipole moment, calculations showed that the energetic barrier to rotation in the *Z*-rotamer relative to the corresponding *E*-rotamer, and then on to cyclization, were lower in these solvents. Along the cyclization pathway, the net dipole moment of each rotomeric species increased due to rotation prior to cyclization (Fig. 24). This energetically favored stabilization can be attributed to the large dielectric energy constant of water. Additionally, the volume occupied by the molecule decreases in going from the *Z*- to *E*-rotamer, *en route* to the lactone. The high cohesive energy, and hence, the difficulty in generating a large cavity in water, can also explain the positive effect on the reaction's overall conversion.

### (f) Miscellaneous reactions

**Fig. 25:**
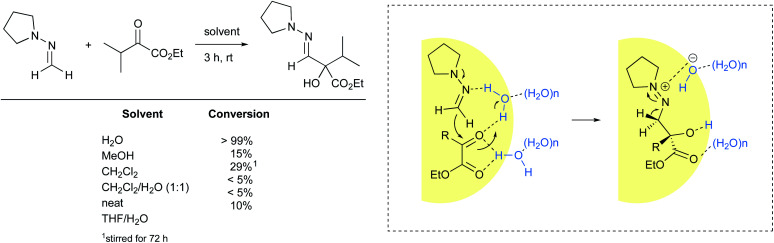
Nucleophilic addition of formaldehyde *N*,*N*-cyclopentylhydrazone to ketones accelerated “on water”.

Nucleophilic addition of formaldehyde *N*,*N*-dialkylhydrazones to α-keto esters is another example of a reaction accelerated “on water”. Under these conditions, the reaction reached 99% conversion after three hours at room temperature. When performed neat or using homogeneous conditions, either in organic solvents or as an aqueous mixture, high levels of conversions were not observed (Fig. 25).^98^ These results suggest that interfacial water molecules figure prominently in the mechanistic pathway. The authors postulated that water brings the two substrates in close proximity while activating the ketone. Carbon–carbon bond formation may happen concurrent with loss of a proton from water, leading to a diazonium hydroxide intermediate in the rate-limiting step. Such zwitterion formation would be stabilized by electrostatic interactions before undergoing deprotonation of the methylene group to afford the desired product. The cleavage of at least one H–OH bond is supported by an observable isotope effect (H_2_O: *t*_1/2_ = 82 min; D_2_O: *t*_1/2_ = 140 min).

### (g) Reactions using organocatalysis

The emergence of organocatalysis has allowed for many transformations which obviate the need for environmentally egregious transition metal catalysts in favor of greener alternatives. Organocatalysts are usually nontoxic, can be easier to dispose of or recycle, and are usually less sensitive to water or air compared to their metal-containing counterparts. Several different types of organocatalysts have been developed. One major category involves covalent activation and bond formation between the catalyst and substrate, as in enamine- and iminium ion-based reactions.^99^ Another classification involves noncovalent interactions between substrates and organocatalyst, such as hydrogen bonding or halogen bonding. The design of highly water-soluble catalysts also opens up new opportunities in this field. Common strategies for increasing water solubility include adding tertiary amines,^100^ amino acids,^101^ or carboxylic acids^102^ to the structure of organocatalysts.

#### (i) Organocatalysis “on water”

Traditionally, the regioselectivity of aldol reactions involving ketones with two distinct α-protons arises from conditions that favor formation of either the kinetic or thermodynamic enolate. For reactions catalyzed by hindered bases, especially at low temperatures, the enolate at the less substituted α-carbon prevails, leading to the kinetic product. Conversely, acid-catalyzed reactions at higher temperatures proceed *via* the enol tautomer, thus the more stable, highly substituted double bond dominates affording the thermodynamic product. However, these preferences are no longer entirely valid when water is involved. In 2010, Gong and co-workers reported that, under otherwise identical conditions, asymmetric aldol reactions between hydroxyacetone and aryl aldehydes in THF favored the *vic*-diol, whereas aqueous conditions favored the 1,4-diol (Table 8). Theoretical studies revealed that water controls the regioselectivity by forming hydrogen bonds with the proline amide oxygen catalyst and the hydroxyl group of hydroxyacetone.^103^

**Table 8:** Use of water to control regioselectivity in organocatalyzed aldol reactions

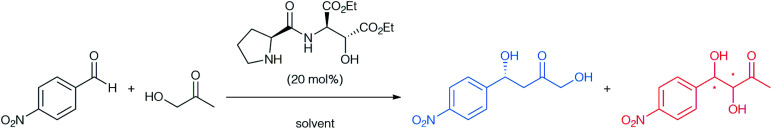				
Entry	Solvent	Yield (%)	ee (%)	Yield (%)
1	THF/water (1 : 0.5)	95	95	<5
2	THF	36	97	58

**Fig. 26:**
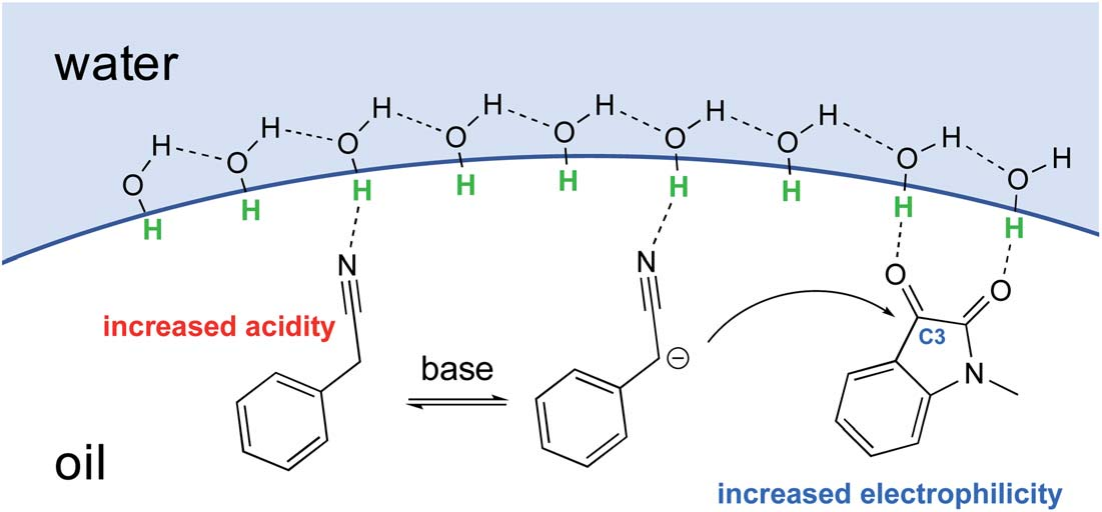
Hydrogen bond activates phenylacetonitrile and *N*-methylisatin.

With conceptualization of the “on water” effect by Breslow and Sharpless,^5,8^ hydrogen bond-containing networks at the water–organic interface are recognized as the main driving force for the high efficiency of several processes in water. The effect of molecular organization has also been applied to aldol-type cyanoalkylation. The interfacial hydrogen bonding increases the acidity of phenylacetonitrile, while increasing the electrophilicity of its reaction partner (Fig. 26).

Thus, the free hydroxyl groups at the interface effectively activated the reactants and stabilized the transition state. When dichloromethane, acetonitrile, MeOH, or DMSO was used as solvent, a significant amount of the undesired dehydration product was observed, while dehydration was completely suppressed using water as reaction medium (Table 9).^104^ The interfacial hydrogen bonding between water and the dicarbonyl group of *N*-methylisatin facilitated this process, as shown by Jian and co-workers using ^13^C NMR experiments. For example, the C3-position in *N*-methylisatin showed a downfield shift from 158.95 to 159.05 ppm.

**Table 9:** Solvent effect on the cyanomethylation of *N*-methylisatin

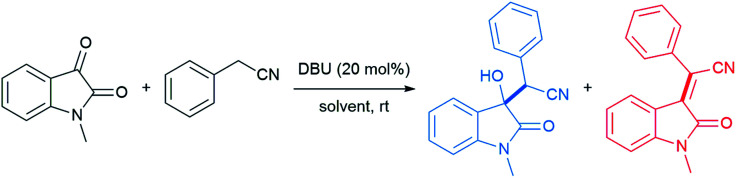				
Entry	Solvent	*t* (h)	Yield (%)	dr (%)
1	DCM	48	9/67	nd
2	Acetonitrile	48	9/64	nd
3	Methanol	48	Traces/92	nd
4	DMSO	48	66/28	65 : 35
**5**	**Water**	**4**	**96/0**	**98** **:** **2**

Moreover, Han *et al.* showed, after reviewing solvent optimization experiments, that protic solvents better facilitated the aldol reaction. Therefore, the evidence seems to point to the conclusion that hydrogen bonds formed between H_2_O and Lewis basic carbonyl groups, such as that present in isatin, result in an enhancement in electrophilicity (Table 10).^105^

**Table 10:** Screening solvents of different strength of hydrogen bonds for an aldol reaction (entries in order of increasing hydrogen bond strength)

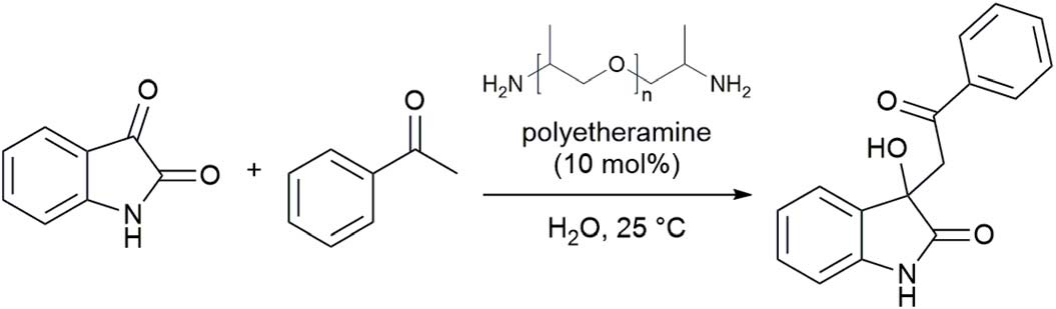		
Entry	Solvent	Yield (%)
1	Hexanes/THF/MeCN	0
2	MeOH	17
3	EtOH	20
4	CF_3_CH_2_OH	29
5	Water	95

Jung and co-workers demonstrated that “on water” conditions influenced by hydrophobic effects can lead to confined transition states that strengthen interactions between chiral catalyst and substrates, ultimately affording higher enantioselectivities.^106^ Described is an investigation into the Mannich reaction of an *N*-Boc protected imine with 2,4-pentanedione, using 1 mol% natural (+)-cinchonine (**CN-1**) as catalyst at room temperature (Table 11). The first evidence for chirality amplification due to the hydrophobic effect was obtained by replacing dichloromethane with brine as the reaction medium. This led to an increase in ee from 22% to 55% (Table 11, entries 1–2). Under biphasic conditions with *o*-xylene, changing brine to LiClO_4_ (aq), which is considered an anti-hydrophobic agent, the ee value dropped significantly from 84% to 15% (Table 11, entries 3 and 4). These results suggest water can induce amplification in resulting product chirality by hydrophobic hydration effects. Under “on water” conditions, the interfacial hydrogen bonds surrounding the confined hydrophobic cavities of microdroplets ensure high proximity of the catalyst and substrates. The impact of the hydrophobic hydration effect on enantioselectivity is further enhanced by decreasing droplet size, which can be achieved by accelerated stirring. Proper magnetic stirring has been known to increase the interfacial area and hence, the hydrogen bonding networks align more efficiently;^107^ the higher the stirring rate the lower the droplet size in the emulsion. Rates of stirring at 200, 600, and 1150 rpm were tested, with maximum ee being reached at 1150 rpm for all six substrates. The effect of the droplet size on enantioselectivity was further elucidated by biphasic microfluidic techniques wherein size-controlled static droplets were generated in microfluidic tubing. After the tube was filled, the two ends were sealed and kept for 24 h without disturbance. The static droplets again verified that smaller droplet volume gave higher ee values. In the confined space of droplets, the strengthened hydrogen bonding on the microdroplet surface forms a water cage and hydrophobic organic solutes inside the hydration shell are more confined and pressurized, leading to more compact transition states, thereby increasing enantioselectivity. The chirality amplification was also observed when high pressure was applied, as this further compresses the transition state.^83^

**Table 11:** Hydrophobic chirality amplification effect

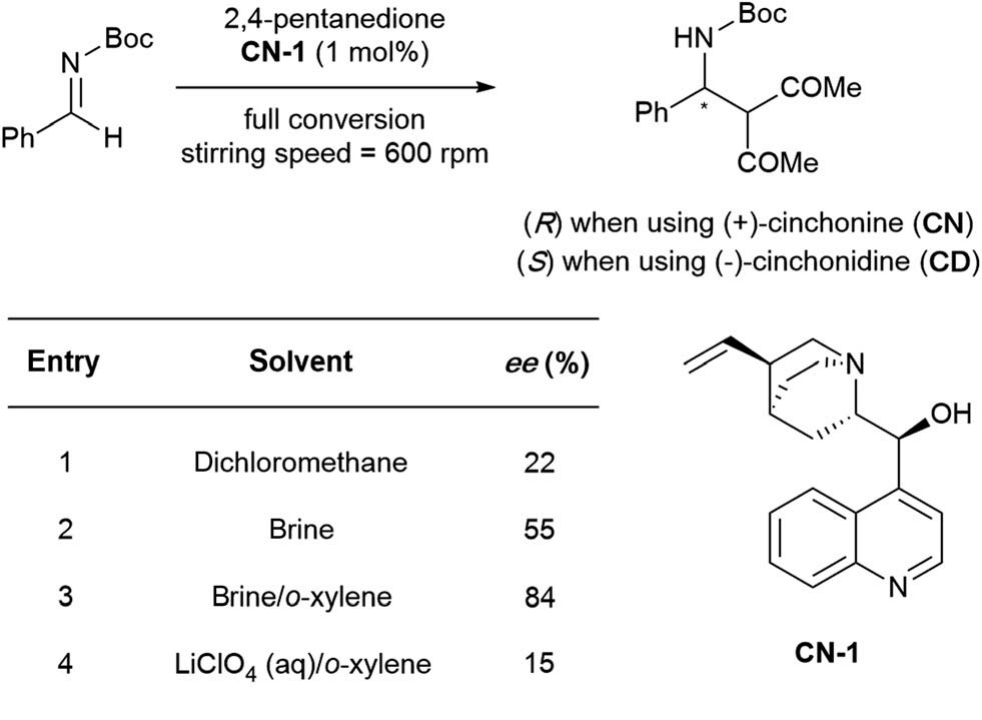

Also in this aqueous medium, catalyst hydrophobicity, on which its affinity to the substrates depends, was tested regarding its impact on amplification of product enantioselectivity.^106^ Two series of catalysts derived from (+)-cinchonine (**CN**) and (−)-cinchonidine (**CD**) were synthesized, with different degrees of lipophilicity associated with their substituents. Though no effect on reaction rate was observed, the same trend of chirality amplification was reported in both series when the more hydrophobic catalysts (higher log *P* values) were used. For **CN**-catalysts, the improvement went from 84% to 96% ee, while the ee's using **CD**-catalysts jumped from 72% to 92% (Table 12). By contrast, the same reactions carried out in dichloromethane gave much lower enantioselectivities (10–24% ee) regardless of catalyst structure.

**Table 12:** The influence of catalyst hydrophobicity on enantioselectivity

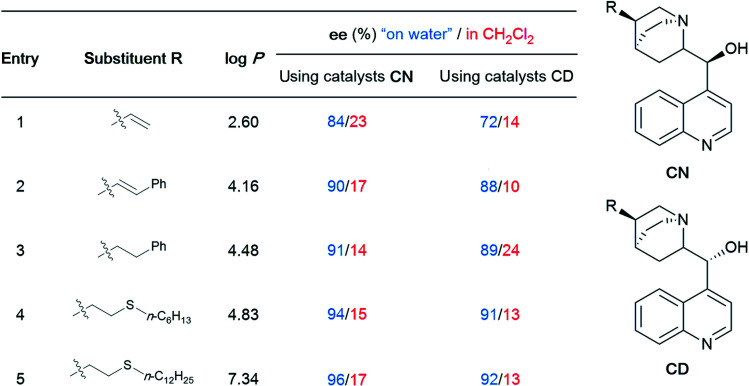

#### (ii) Organocatalysis in micellar media

**Fig. 27:**
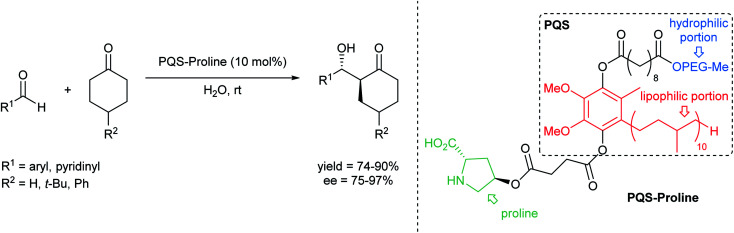
PQS-Proline-catalyzed aldol reaction.

A major drawback to organocatalysis is the potential need for high loadings of catalyst (*e.g.*, see Table 8, above), which oftentimes are not recovered and recycled. The pharmaceutical industry, given its notoriety surrounding the large E-factors associated with drug syntheses,^108^ increasingly looks for opportunities to recycle all components of reaction mixtures to the maximum extent possible. In this regard, the design of reusable catalysts has gained momentum.^109^ One approach aimed at using water as the reaction medium focuses on asymmetric aldol reactions, where the organocatalyst is covalently bonded to the surfactant.^110^ This option to attach a proline group is made possible by using a hydroquinone, in this case derived from the dietary supplement ubiquinol (the reduced form of coenzyme Q_10_), where each hydroxyl group allows for functionalization. Thus, after conversion to the corresponding surfactant (*via* esterification with MPEGylated sebacate linker; *i.e.*, “PQS”), subsequent attachment of proline (*via* reaction of its 3-hydroxy group with succinic anhydride) leads to “PQS-Proline”, which forms nanomicelles upon dissolution in water. The proline moiety is forced to remain within the micelle's inner lipophilic core, in close proximity to the reactants with only water as the surrounding medium. Enantioselective aldol reactions were then performed at room temperature (Fig. 27). Workup simply involved extraction of product from the aqueous mixture, leaving behind the catalyst which was reused and exhibited the same reactivity over four cycles. This method out-performs those with proline immobilized on solid supports.^111^

Another example of an asymmetric aldol reaction-oriented amphiphilic organocatalyst was developed by Qin *et al.*^112^ In contrast to PQS-Proline, Qin's amphiphile, **PTC12**, utilizes the catalytic portion as the sole hydrophilic moiety rather than MPEG (Table 13). As a result, when dispersed in water, the catalyst is necessarily oriented outward from the emulsion droplets toward the aqueous media, and thus water plays a more direct role in catalysis. On its own, the surfactant is not soluble in water, so compressed CO_2_ is employed to aid in dissolution by forming carbonic acid in the aqueous medium. This protonates the amine of the catalyst and forms the bicarbonate salt, thereby increasing the hydrophilicity of the headgroup and promoting self-assembly into nanostructures (vesicles). In addition to solubilizing the amphiphile, compressed CO_2_ dissolves into the lipophilic core of the vesicles and increases their size, allowing for regulation of the microenvironment around the dispersed nanoparticles.^42^ Both yields and enantioselectivities improved as the pressure increased from 0 to 5 MPa (Table 13, entries 1–4), which was attributed to the increased size of the nanoparticles, leading to a greater number of available catalytic sites. As pressures increased from 5 to 8 MPa, however, a sharp decrease in ee was noted with no appreciable decrease in yield (entries 4–6), indicating that the curvature at the interface plays a crucial role in promoting stereoselectivity by stabilizing the transition state. A marked improvement in both yield and enantio-selectivity was observed when the medium was switched from pure water to water saturated with NaCl (entry 7) owing to the increase in hydrophobic interactions, *i.e.*, salting-out, and the resulting increase in local substrate concentration inside the lipophilic spaces within each vesicle. When the reaction was performed with l-proline instead of the amphiphilic organocatalyst, under otherwise identical reaction conditions, no conversion to products was observed (entry 8), indicating that self-assembly of the organocatalyst into nanostructures, thus the presence of the interfacial microenvironment, were essential for product formation.

**Table 13:** Effect of pressure, salinity, and nature of the organocatalyst on asymmetric aldol reactions

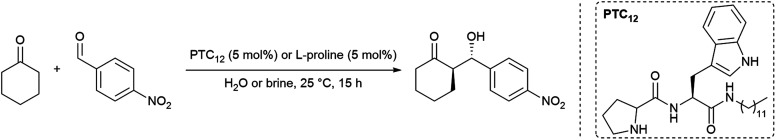						
Entry	Catalyst	Medium	*P* (MPa)	Yield (%)	*Anti*/*syn*	ee (%)
1	**PTC12**	H_2_O	0	0	—	—
2	**PTC12**	H_2_O	2	81	81/19	37
3	**PTC12**	H_2_O	4	92	82/18	39
4	**PTC12**	H_2_O	5	94	81/19	51
5	**PTC12**	H_2_O	6	93	80/20	48
6	**PTC12**	H_2_O	8	93	83/17	28
7	**PTC12**	Brine	5	99	84/16	93
8	l-Proline	H_2_O	5	0	—	—

**Fig. 28:**
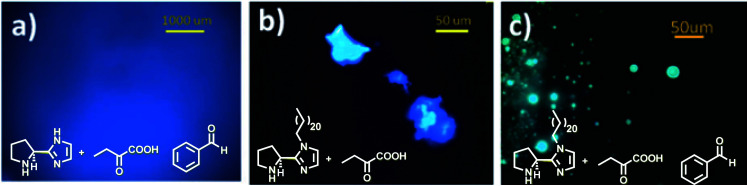
Fluorescence microscope images of reaction mixtures in presence of (a) non-amphiphilic catalyst and α-ketoacid in water, (b) amphiphilic catalyst and α-ketoacid in water, (c) and amphiphilic catalyst and both starting materials in water.

Zhang *et al.* developed an amphiphilic 2-pyrroloimidazole organocatalyst for the synthesis of chiral isotetronic acids from aldehydes and α-ketoacids in water (Table 14).^113^ As with Qin's **PTC12** amphiphile,^112^ Zhang's surfactant (entry 2) uses the pyrrolidine portion as the sole hydrophilic moiety, thus forcing reactions to occur at the “oil”/H_2_O interface of the micelles. This proved crucial for obtaining high conversions and enantioselectivities, as demonstrated by the reactions, in water, of substrates with an organocatalyst lacking a greasy hydrocarbon tail (entry 1). The catalyst, with R = H, dispersed homogeneously into aqueous solution (as illustrated by fluorescence spectroscopy; Fig. 28a) but led to both poor conversion and stereoselectivity (entry 1). By contrast, the same reaction with the amphiphilic catalyst led to heterogeneously dispersed droplets (Fig. 28b and c), and dramatically improved conversion and ee (entry 2).

**Table 14:** Effect of catalyst amphiphilicity on the conversion and stereoselectivity in the synthesis of isotetronic acids

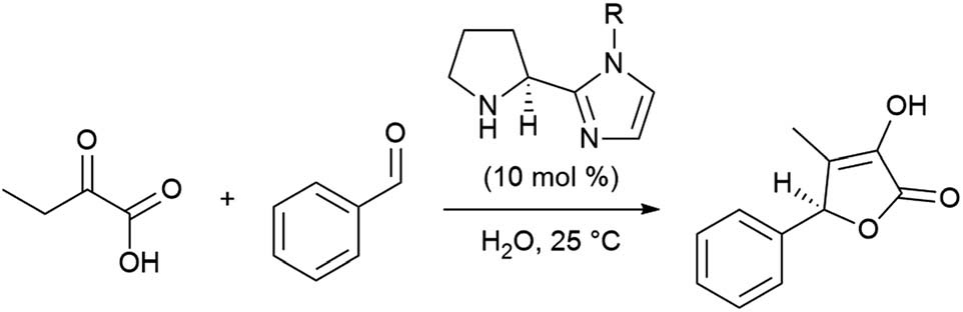				
Entry	R	*t* (h)	Conversion (%)	ee (%)
1	H	144	8	64
2	*n*-C_22_H_45_	24	95	94

**Fig. 29:**
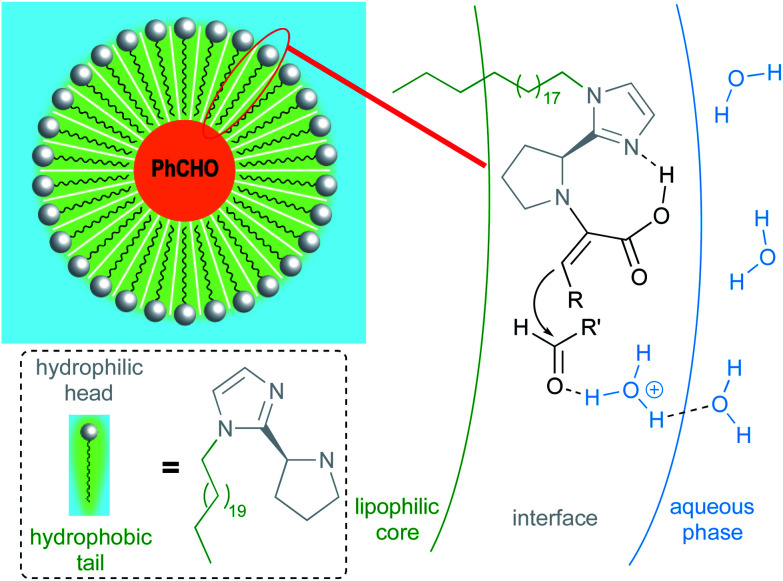
Enlarged diagram of emulsion droplets, interfacial region, and proposed reaction model.

The proposed mechanism for the water-enabled reaction is illustrated in Fig. 29. The α-ketoacid is activated by the catalyst headgroup *via* enamine formation also involving hydrogen bonding of the imidazole to the acid moiety. The lipophilic aldehyde is housed inside the core of the micelle, allowing only the aldehyde moiety to protrude into the interfacial region. The aldehyde carbonyl is then activated by dangling –OH groups protonated by the α-ketoacid. The high local concentrations of substrates at the interface also contribute to both high conversions and enantioselectivities.

#### (iii) Organocatalysis in vesicles

**Fig. 30:**
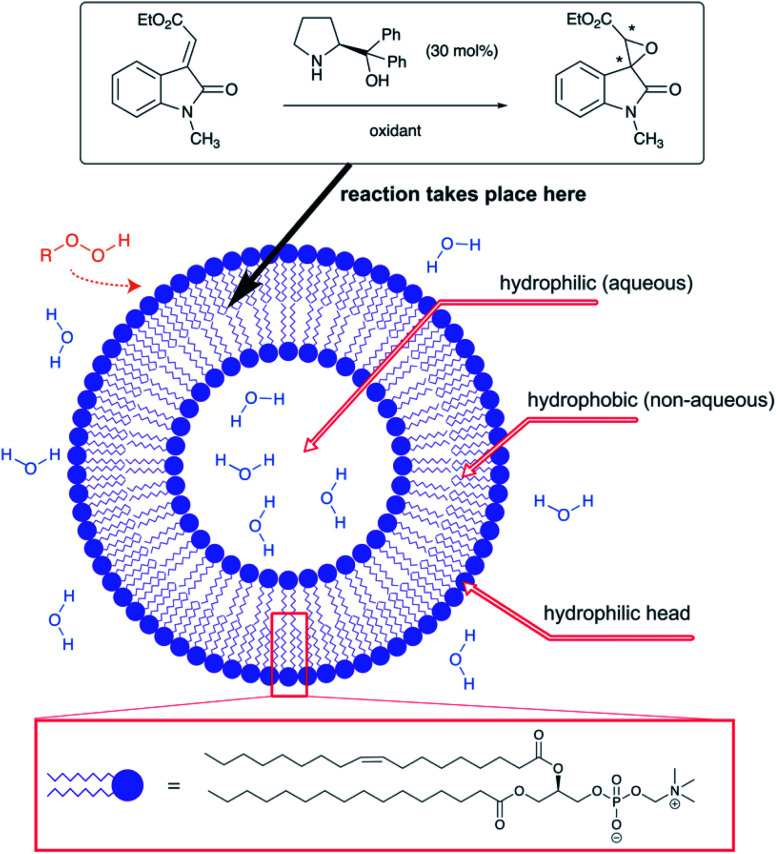
Organocatalyzed epoxidation of an α-alkylidene oxindole in aqueous liposomes.

Liposomes, being one form of a vesicle, offer yet another soft and dispersed interface-rich aqueous system that can offer useful results in organic transformations. As with nanomicelles, these bilayered spheres, composed of phospholipids, can accommodate organic media-soluble substrates and catalysts within their lipophilic rings (rather than within micellar inner cores). They offer the advantage of a more controlled environment given their precise packing geometry. Moreover, the kinetics associated with lipid exchange, as opposed to surfactant exchange, is slower. Thus, as illustrated in Fig. 30, this ordered environment in water allowed for stereoselective epoxidation of α-alkylidene oxindoles, assisted by a prolinol organocatalyst.^114^

### (h) Photoreactions “on water”

**Fig. 31:**
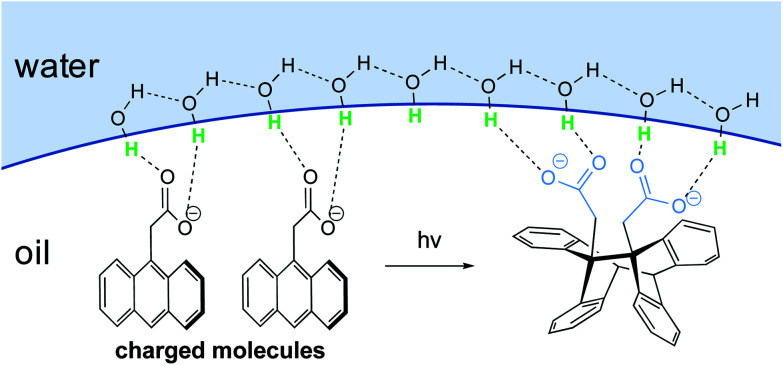
Substrate orientation at the “oil”/water interface.

An example of a light-initiated reaction involving “on water” conditions includes irradiation of 9-substituted anthracenes (Fig. 31). Cycloaddition of the aromatic rings at the 9 and 10 positions yields head-to-tail (h–t) or head-to-head (h–h) photocyclomers. Tung and co-workers^115^ revealed an interesting regioselectivity attributed to “oil”/water interactions. When the anthracene bears a polar or charged functional group, such as –CH_2_N^+^(CH_3_)_3_Br^−^, –CH_2_COO–Na^+^, or –CH_2_OH, the substituent orients itself towards the water phase. On the other hand, the hydrophobicity of the anthracene moiety forces the chromophore to remain in the organic phase. Thus, the anthracenyl plane would lie perpendicular to the interface of the surrounding water. This pre-orientation favors formation of the h–h adduct >90%, while the increase in local concentration at the interface raises the quantum yield of the photocycloaddition. Alternatively, reactions performed in organic solvent, such as dichloromethane, result in mixtures of head-to-head and head-to-tail cycloaddition products, with the head-to-tail isomer predominating. This regioselectivity was justified in terms of the electrostatic and steric effects of the substituents, R, at C-10 on the anthracene ring. For the relatively less polar groups such as acetyl (COCH_3_), the ratio of h–h : h–t in water drops to 62 : 38, resulting from a less organized alignment. For the nearly non-polar case of R = CH_3_, essentially no difference is observed between reactions in dichloromethane or water since there is no apparent driving force to establish a preferred substrate orientation (Table 15).^87^

**Table 15:** Effect of substituent polarity on product distribution (entries in order of decreasing polarity)

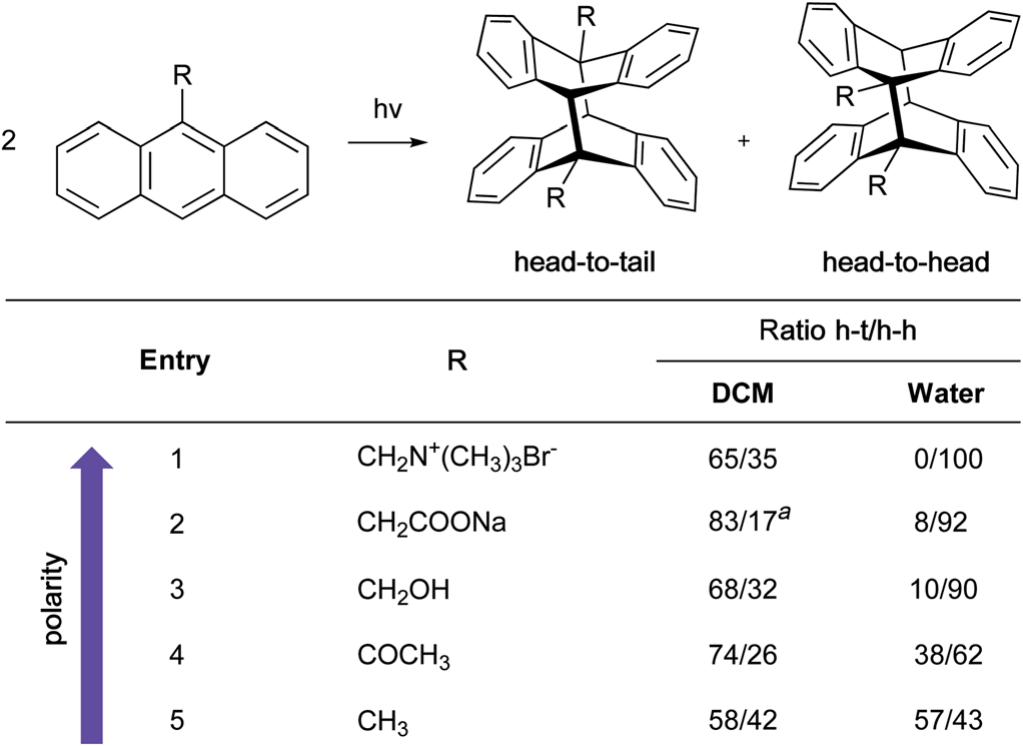

a Ratio in diethyl ether.

## 5. Conclusions

Organic chemistry in water is still in its infancy, especially when thought of in terms of “chemo-catalysis”. But such is also the situation from the “bio-catalysis” perspective, as directed evolution continues to develop new non-natural enzymes that work their magic … *in water*. While organic solvents are likely to be around for years to come, the toolbox for organic transformations in water is growing exponentially, providing a wealth of opportunity to move away from chemistry in petroleum-based media and towards water as the bulk reaction medium.^116^ However, the reality is that most of that toolbox, as of today, remains empty. Indeed, one could argue that most, if not all, of the fundamental organic chemistry presented in any modern text is severely dated and in need of “greening”. But still, no textbook written from the green chemistry perspective exists. Broadly viewed, areas *e.g.*, reduction chemistry and enolization, to name only two that feature prominently in sophomore organic chemistry classes, are destined to be upgraded with sustainability in mind. Reagents such as DIBAL and LAH, introduced many decades ago, are examples of especially valuable sources of hydride but that require unforgiving conditions of dry organic solvents and careful temperature control, two parameters that disappear when the medium is water. Surely, we can develop more modern reagents that achieve the same ends in aqueous media. Nature already has the equivalents, assuring us that this is very doable. And as for a general discussion of carbonyl chemistry under the influence of a strong base, do we really need such environmentally egregious LDA in THF at −78 °C? Are we not clever enough to find alternatives in water, as Nature did eons ago using, *e.g.*, aldolases? Si se puede!

Over the years, water has been a suitable “solvent” for selected transformations in organic chemistry despite pre-conceived notions of its inappropriate dissolution capabilities. In many cases, it has outperformed traditional organic solvents due to its unique properties. Indeed, while water is the perceived enemy of reactions involving Grignard, organozinc, and even organolithium reagents, recent literature disproves such outdated, parochial thinking.^94,117,118^ And while the debate remains regarding the prospects for switching^119^ numerous reactions from organic to aqueous media, there is no argument that water can play varying roles beyond that of the gross reaction medium. This review highlights some of the unique properties that can impact reaction outcomes, and which salient features to consider when designing systems for water-based chemistry. The discussion of “on water” chemistry applies to reactions with a negative volume of activation (*e.g.*, cycloadditions, condensations, and multicomponent reactions). The high cohesive energy density and clathrate formation influence stereoselectivity as well by forcing formation of tighter transition states. In the presence of H-bond acceptors within reactants, the activating ability of dangling –OH groups at the interface is another distinctive aspect leading to faster transformations, especially if the Lewis basicity of a reactant is relatively high. The importance of nanomicelles that serve as nanoreactors dissolved (or aggregated) in water, relative to “in water” (implying substrate/catalyst solubility in this medium) or “on water” (indicating total insolubility of substrates/catalyst in water) can be easily tested by performing the reaction in the absence of a surfactant. In most cases (except for reactions that readily take place “on water”), the background reaction takes place to varying extents, but rarely with the same levels of conversion and ultimately, yields. Many of the synthetic benefits associated with using nanomicelles in water remain to be discovered, as only a few have been disclosed of late, including the “reservoir effect”,^59^ lipophilic ligand design,^38,120,121^ and the “nano-to-nano” effect.^44^ Clearly, when water is intimately involved, reactions can be governed by different “rules”.^43^ Ultimately, the aqueous medium containing additives that enable chemistry in water must be treated as “waste water”. Recycling aside, the community must ultimately deal with issues such as toxicity and biodegradability, associated with, *e.g.*, each surfactant. But again, significant progress on this front is being made, and as interest grows in performing reactions in aqueous media,^122,123^ these issues will be solved while new and exciting phenomena will emerge. Water's status as the synthetic chemist's best friend is inevitable, as chemistry in water is our future.

## Conflicts of interest

The authors declare no conflict of interest.
